# Intertidal fishes of Mauritius with special reference to shallow tidepools

**DOI:** 10.3897/BDJ.7.e36754

**Published:** 2019-08-06

**Authors:** Erik Arndt, Ronald Fricke

**Affiliations:** 1 Anhalt University of Applied Sciences, Bernburg, Germany Anhalt University of Applied Sciences Bernburg Germany; 2 Im Ramstal 76, Lauda-Königshofen, Germany Im Ramstal 76 Lauda-Königshofen Germany

**Keywords:** Teleostei, Western Indian Ocean, intertidal residents, tidepools, ecology

## Abstract

Intertidal fishes are found in large numbers and play an important role in their ecosystems, but knowledge of their ecology is still very limited in many tropical regions. Within this context, data from intertidal fishes in Mauritius were compiled from different sources and intertidal resident species were examined in Mauritian tidepools. A total of 292 fish species occurring in Mauritius were reported from intertidal habitats, of which 62 species represent permanent intertidal residents. The species number in the studied pools increased, not only with the proportion of stones and rock covering the pool bottom, but also with pool facilities, for example, the supply of boulders and a high coverage of macro-algae. All examined pools were dominated by two species, *Bathygobius coalitus* and *Istiblennius edentulus*. Their abundance increased with decreasing pool size, peaking in pools with a surface area between 1-2 m^2^ during the lowest level of ebb tide. This 'overcrowding effect' may be linked to the absence of predators in these very small pools. The comparison of present data with results of a survey made in the same area in 1995 suggested a decrease of resident species occurred during the last decades, probably linked to human influences, such as eutrophication and water pollution.

## Introduction

Intertidal species occupy the narrow band of near-shore habitats between the tidemarks of seas and oceans (Horn et al. 1999). Fishes occurring in the intertidal zone represent numerous different families and constitute a rather inhomogeneous group. Some of these fishes visit the near-shore habitats only briefly, mainly to forage. Others inhabit the intertidal zone during part of their life cycle, for example, the juvenile phase, whereas a third group of fishes, having highly specific adaptations in terms of behaviour and physiology, spends their entire life in intertidal habitats (Horn et al. 1999, Griffiths 2003a).

In a first overview of intertidal fishes, Chotkowski et al. (1999) recorded 702 species representing 110 genera in one chondrichthyan and 20 teleost families. This large number of species is based mainly on the North Atlantic, North-eastern Pacific, South African and New Zealand regions. Chotkowski et al.'s review included only one study each from the Western Central Atlantic, Indian Ocean, subtropical Australia, Central Pacific and South-eastern Pacific and three studies from the tropical Eastern Pacific. The level of knowledge about intertidal fish communities in many tropical regions, for example, in the tropical Eastern Atlantic and Western Indian Ocean, has hardly changed since then. Possible reasons for the limited knowledge are the low importance of intertidal fishes for fisheries, their occurrence in extremely shallow waters, their small size and their sometimes secluded way of live. The species in question are rarely recognised by divers and hardly considered by shore visitors.

On the other hand, intertidal fishes are found in large numbers of species and may reach high abundances. Based on their quantitative occurrence, it can be inferred that they make an important contribution to the intertidal food web by functioning in their roles as herbivores or predators. Though quantitative analyses of food webs including intertidal fishes are still scarce, we know that the diversity of herbivores is higher in tropical zones than in temperate regions and that intertidal fishes may have a strong influence on algal diversity and algal abundance in temperate as well as tropical areas (see Horn and Ojeda 1999 for a summary). For example, herbivorous fishes accounted for 20-30% of the fish communities in temperate rocky reefs of Australia (Jones and Andrew 1990) and 20% of total intertidal fish abundance (or 51% of total fish biomass) in temperate Chile (Stepien 1990), while 81.6% of the fish biomass at a subtropical intertidal reef in South Africa consisted of herbivorous fishes and omnivorous fishes with a notable herbivorous portion of their diet (Berry et al. 1982). Thus, intertidal fishes play an important role in material turnover in their ecosystems (Bennett et al. 1983). More detailed knowledge on these communities would provide a deeper insight into the ecology of marine shallow water environments. Since tidepools, estuaries and other intertidal habitats are located nearshore, they are vulnerable to human impacts such as eutrophication, pollution, disturbance or destruction. Therefore, better knowledge and monitoring of intertidal fishes would open new opportunities in applied ecology, for example, by using these fishes as ecological indicators for environmental pollution and climate change.

Against this background, one aim of the present study is to make a first compilation of data on fish species occurring in the intertidal zone in Mauritius. A second aim is the examination of intertidal resident species in Mauritian tidepools. The results of this study will increase our knowledge on intertidal communities in the Western Indian Ocean and could inspire further research towards use of intertidal species for monitoring and applied approaches in environmental research.

## Material and Methods

### Study area

**Figure 1.**
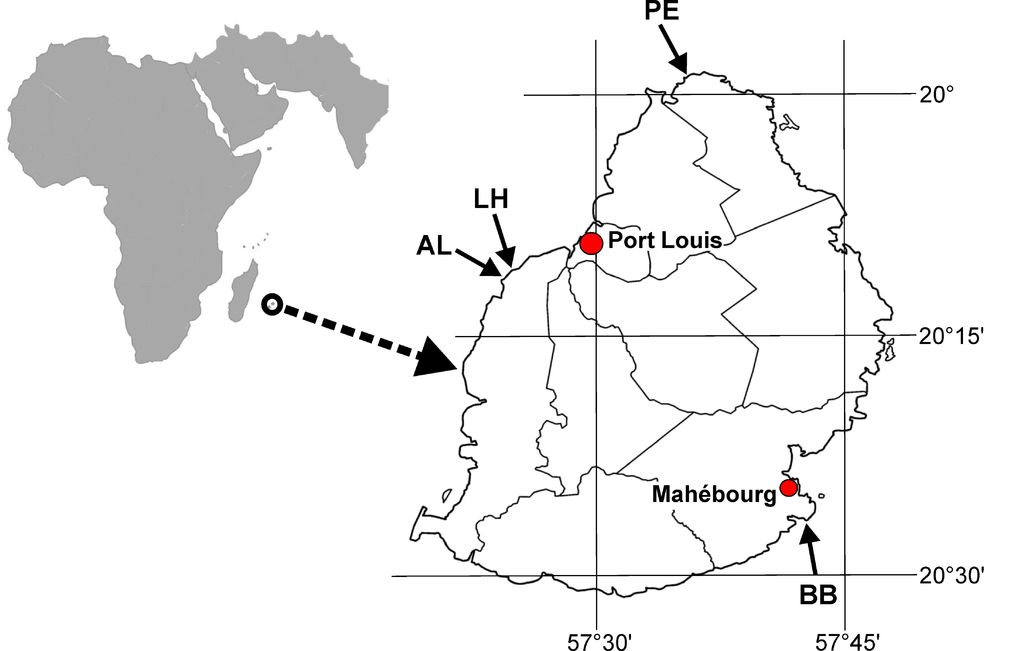
Location of Mauritius in the western Indian Ocean (left) and examined tidepools in Mauritius (right). AL – Albion; BB – Blue Bay; LH - Lighthouse at Pointe aux Caves, north of Albion; PE – 1 km north-east of Péreybère public beach.

The island of Mauritius is located in the Western Indian Ocean (Fig. 1), 800 km east of Madagascar between 19.58°S and 20.31°S, as well as between 57.18°E and 57.46°E. Together with Réunion, Rodrigues and several smaller islands, it forms the Mascarene islands. Mauritius has a coastline of 322 km (Duvat 2009) and is surrounded by a submarine platform, allowing the development of a fringing coral reef with a broad lagoon that protects much of the coastline in the north, east, south-west and north-west (Pichon 1971). The fringing reef is disrupted by rocky shores and cliffs in the south and west. Sea surface temperatures vary seasonally, with a minimum of 22°C in August and reaching up to 29°C in February. The dominant current patterns are from east to west, influenced by the south-eastern trade winds, which are stronger during the winter months than during the summer (McClanahan et al. 2005, Ragoonaden 1997). Tides occur semi-diurnally, with a mean tidal range of 0.9 m during springtime and 0.1 m during neap time (Montaggioni and Faure 1997).

Despite its small size, Mauritius has a large diversity of coastal habitats. Besides sandy beaches, mostly connected to the lagoon, there are estuaries of 51 rivers and streams, which are often connected to mangrove areas formed by the two rhizophoracean species *Bruguiera gymnorrhiza* (L.) Savigny and *Rhizophora mucronata* Lam. (Fagoonee 1990). Coastal parts, directly exposed to wave action, reveal basaltic cliff boulders. The latter coastal sections comprise a number of tidepools that represent, together with estuaries and mangrove areas, the most common intertidal habitats.

### Examined pool sites

**Figure 2.**
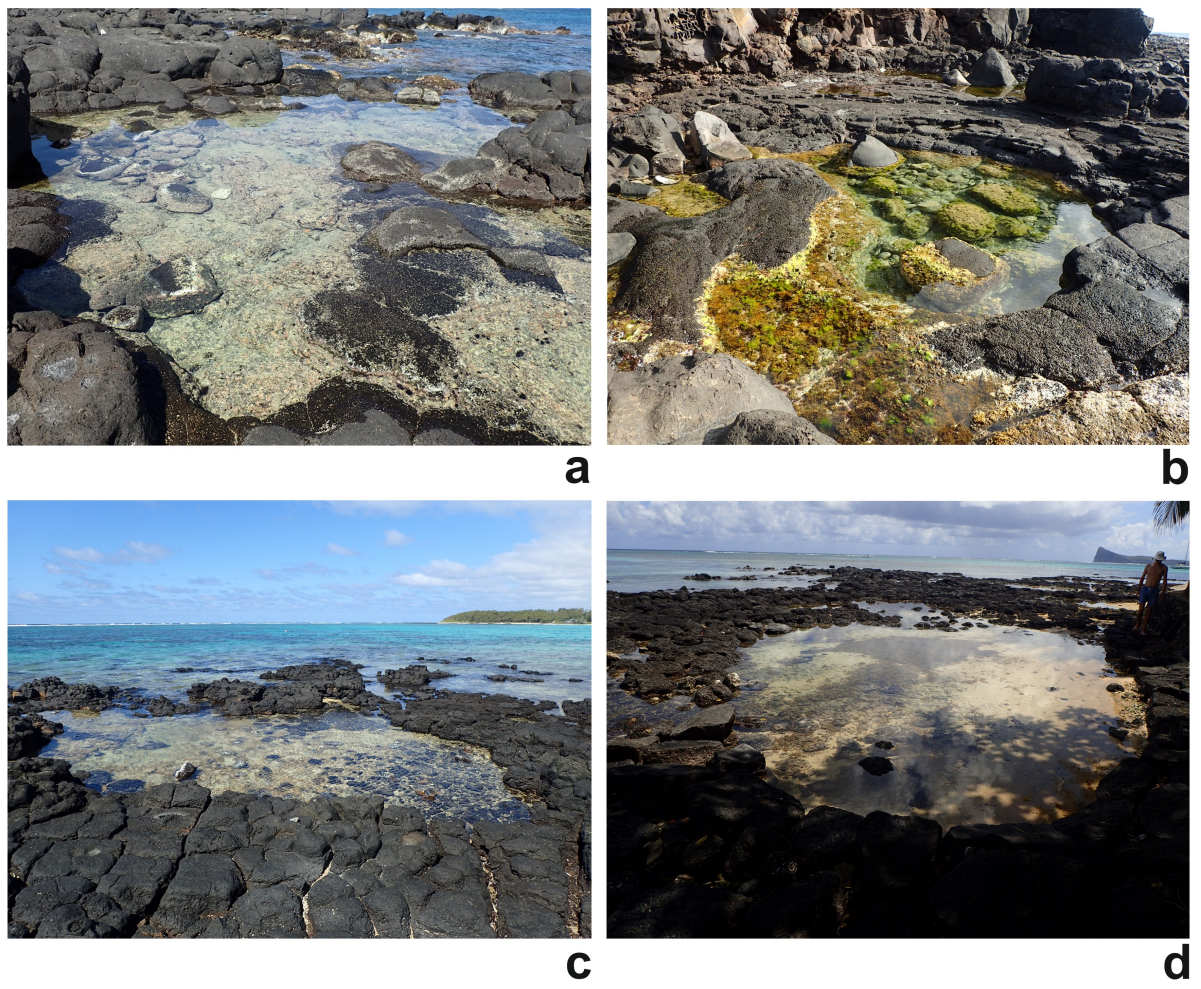
Examples of examined tidepools in Mauritius. **a** – ALD6 in Albion, exposed to the open sea; **b** - LH1, located on a rock platfom below the lighthouse at Pointe aux Caves; c – BB1 in Blue Bay, located inside the lagoon; d – PE1 near Péreybère, located inside the lagoon.

Five groups of tidepools were examined in four localities of Mauritius (Fig. 2, Suppl. material 1). The pools around Albion (AL) and below the lighthouse at Pointe aux Caves (LH) directly face the open sea and are thus exposed to stronger wave action. At these sites, the water streams in with more physical power during high tides. The tidepools in Blue Bay (BB) and in Péreybère (PE) are located within the shallow lagoon (depth 0.6-2.0 m) and most of them were characterised by a low to very low diversity, combined with a very high evenness (Suppl. material 1). In these areas, nearshore platforms of lava tuff substrate form shelves with crevices and pools, resulting in ranges of tidepool habitats. During flood and ebb, the water streams moderately in and out of the pools, which are mostly characterised by sandy bottoms.

The pools were visited first in September/October 2017 and once more in September/October 2018. The following characterisation of tidepools refers to the condition in September/October 2018. Information concerning surface area and depth refers to the condition at lowest level of ebb tide.

**Albion (AL).** From the northern point of the public beach onwards, the north-western region of Albion includes a rocky coast with numerous tidepools. Two groups of tidepools were examined, including six pools close to the end of Avenue des Dattiers (in several maps erroneously referred to as Ave. des Rossiers) and five pools close to the end of Avenue St. Géran. The latter site is located 400 m south-east from that at the end of Avenue des Dattiers.

The pools close to the end of Avenue des Dattiers comprised three small (6-10 m^2^) and three larger basins (20-40 m^2^ at the lowest level of ebb tide) with a depth of 25-50 cm, where area and depth did not correlate. The bottom substrates resembled those in the pools at Avenue St. Géran except in two of the smaller pools, which had rocky bottoms only. One of these pools was permanently streamed through by seawater, the remaining five were isolated except for a short period during the highest point of flood tide. The southernmost of these pools is shown in (Fig. 2a).

The pools close to the end of Avenue St. Géran had a surface area of 16-45 m^2^ and a depth of 25-40 cm during the lowest level of ebb tide. Rocky bottom covered approximately 35-80% of the bottom surface. In the two largest pools, there also occurred macro-algae and soft-corals (less than 5% of bottom surface) and tiny green algae representing the potential food of blennies and pomacentrids. The tiny green algae were too small to estimate their ground cover. One of these pools was isolated except during the highest point of flood and two were continuously streamed through by seawater.

The five pools with highest diversity were all located in the vicinity of Albion (Suppl. material 2).

**Pointe aux Caves, below lighthouse (LH).** Pointe aux Caves is characterised by a high rock cliff with two platforms lying one metre above the mean water level (Bhikajee 1996), i.e. a few decimetres above the middle high water level. The northern platform contains three rockpools, two of which were examined (Fig. 2b). The pool surfaces were 28 and 30 m^2^, respectively, the depth 100 and 60 cm. Green macroalgae covered about 30% of the bottom surface in the extended shallow parts of both pools. Due to the high platform, the pools did not have any seawater exchange or connection to the water in the open sea, except at the highest level of flood during spring tides. Nevertheless, a high number of fish (permanent pool residents) inhabited these pools.

**Blue Bay (BB).** A triangular area of flat rocks, consisting of lava tuff, extends at the southern end of Blue Bay, close to the 'Le Peninsula Bay' Beach Resort and Spa. Depending on seasonal water level, the lower parts of this rock flat constitute a number of tidepools. Seven of these tidepools were examined. The surface area of these pools ranged between 1-15 m^2^ during the lowest level of ebb tide and 4-36 m^2^ during flood tide. The maximum depth ranged between 10-25 cm at lowest level of ebb tide. The bottom surface was mostly characterised by sand (50-80% bottom cover) and stones (10-40% bottom cover), while living brown algae occurred in four of the pools (usually with 10-15% bottom cover, but covering 50% of the bottom surface in one of the smaller pools). The largest and westernmost pool is shown in Fig. 2c.

**Péreybère (PE).** Several shallow tidepools are located 1000 m north-east of Péreybère public beach. Three pools were examined; these had a surface area of 36-54 m^2^ and a maximum depth of 15-25 cm during the lowest level of ebb tide. The bottom was covered with sand (70-90% of the bottom surface) and stones and a notable amount of dead brown algae floated in two of the pools. The pools were isolated for less than 2-6 hours during a single tidal cycle. The largest and westernmost pool is shown in Fig. 2d.

### Methods

#### Compilation of data

In order to get a complete list of intertidal fish species in Mauritius, all available literature was searched, extracting data on fishes recorded from intertidal habitats and their localities. Besides the published records from Mauritius (Fricke 1999), data from Réunion (Fricke et al. 2009), Europa Island (Fricke et al. 2013), Glorieuses Islands (Durville and Chabanet 2009) and several further places were also considered for those species that are known from Mauritius but for which published intertidal records from Mauritius were lacking so far. Additionally, material from the following museum collections was included: USNM, Smithsonian Collection, Washington (records by P.C. Heemstra, A.C. Gill, D.G. Smith and M.J. Smale from April/May 1995, see Fricke 1999), SMNS (Staatliches Museum für Naturkunde, Stuttgart, Germany) and BMNH (The Natural History Museum, London). The complete list of species and sources is given in the Results chapter. Family authorships were cited according to van der Laan et al. (2014); genus and species classification follows Fricke et al. (2019).

Particular attention was given to fishes reported from tidal pools, because pools are well-defined intertidal habitats that isolate their communities at least during ebb tides, making observations and examination of fishes relatively easy. In contrast, the border between the tidal and subtidal zones appears to be a smooth transition that is difficult to identify in many other near-shore habitats such as sand flats, rock cliffs or lagoons. Tidepools were also the subject of field examinations in the scope of the present work.

Fishes may occur in the intertidal zone for quite different reasons or in various periods of their life. Therefore, it is necessary to classify different 'types' of intertidal fishes. The definition of these terms follows Thomson and Lehner (1976) and Griffiths (2003a)

R – *permanent residents*, spending their entire life (juvenile to adult) within tidepools or the intertidal zone. They are often highly adapted for intertidal life by possessing specialised behavioural or physiological adaptations.

O – *opportunists* (also *secondary* or *temporary* or *partial residents*), living in the intertidal zone / tidepools during specific life history stages or seasons. They are also widely distributed in the subtidal zone.

T – *transients* (or *tidal visitors*), using the intertidal zone including tidepools transiently for foraging. They may end up accidentally trapped in pools as the tide goes out.

The assignment to one of these categories was established on the basis of literature references such as Murase (2013) and Sindorf et al. (2015), our own observations and information about size as well as developmental stage of the examined fishes in different studies, such as Ntiba et al. (1993), Durville and Chabanet (2009), Ghanbarifardi and Malek (2009) and González-Murcia et al. (2016). It is worth mentioning that there are no generally used abbreviations of the aforementioned categories in the existing literature, for example, PR means partial resident in Cox et al. (2011), but permanent resident in Sindorf et al. (2015).

#### Field examination of fishes in rockpools

The fishes of selected tidepools (see description above) were examined in September/October 2017 and 2018 by underwater visual census, i.e. fishes in each pool were observed and photographed. The main objectives during field work in 2017 were the selection of pool sites and the identification of fish species in these pools. Individuals were not counted during the field work in 2017. In 2018, the pools were examined systematically, i.e. fishes in each pool were observed and registered both near to the lowest level of ebb tide and highest level of flood by day. One additional examination took place in each pool by night. Photos for later analyses were taken of any fish for which a definite field identification was not possible. During the surveys at low tide-level, the number of permanent resident species was counted to estimate their abundance (cf. Gibson 1999). Counting as well as taking photos was feasible, because the pools were shallow and not too large. The permanent resident species showed territorial behaviour or used a limited area of their pools only, all of them being diurnal. Despite a detailed search, seclusive or nocturnal species were not found. During night, the permanent resident species rested motionless on the pool bottom or in small crevices and allowed close-up shots of morphological details. The species could be identified on the basis of photographs taken by day and night. Other species, representing secondary residents or transients, were simply photographed and registered without counting.

#### Statistical analyses

Besides pool resident and transient fishes, several pool parameters were recorded in order to assess whether pools with particular characteristics support a higher number of fish species or fish individuals. The pool parameters are:

- water surface area (during ebb tide; in m^2^)
- pool depth (during ebb tide; in cm)
- isolation of the pool (i.e. the duration within one tidal cycle in which water did not stream into or out of the pool; in % of the whole cycle)
- substrates (sand, stones, pebbles, macro-algae; in % of bottom coverage)
- boulders (in % of bottom coverage)

Spearman rank correlations were calculated to show simple correlations between certain pool characteristics and the total number of species per pool or the number of individuals of a fish species.

Direct gradient analyses (redundancy analysis, RDA) were used to relate the abundance of species to measured pool characteristics (Ter Braak and Šmilauer 2002). As pools of different locations showed different compositions of species (especially with regard to the locations inside the lagoon vs. those on rocky shores without lagoon), two separate RDA calculations were made: (i) including all pools but only the two ubiquitous species *Bathygobius coalitus* and *Istiblennius edentulus* and (ii) including rockpools in the environment of Albion and Pointe aux Caves only, but all of the species recorded at these sites. Furthermore, the species diversity and evenness in the pools were calculated in order to enable a comparison of different pools. We used Buzas & Gibson's evenness and Brillouin's index as diversity measure, which is recommended in the case where nearly the whole community is known and sampling is done without replacement of individuals (Krebs 1999). The indices were calculated using PAST (Hammer et al. 2001).

## Data resources

All primary data collected for this study are available as supplementary files. Suppl. material 1 contains parameters and characteristics of the examined tidepools; suppl. material 2 contains the recorded permament intertidal resident fish species in Mauritian shallow tidepools.

## Results

### Survey of intertidal fishes

A total of 292 fish species from Mauritius were reported from intertidal habitats in literature (Table 1). Amongst these species, there are approximately 55 transients, 175 opportunists and 62 permanent residents. The category of some species, here provisionally listed as opportunists, could not be determined with certainty if not enough data were available.

Moray eels (Muraenidae), gobies (Gobiidae), roundheads (Plesiopidae), damselfishes (Pomacentridae), triplefin blennies (Tripterygiidae), blennies (Blenniidae), kelp blennies (Clinidae), dragonets (Callionymidae) and probably also snake eels (Ophichthidae) include permanent intertidal residents that may spend their whole life in tidepools. This applies to 32 out of the 62 permanent residents in Table 1. A few permanent resident species, including *Springeratus polyporatus* (Clinidae), *Bathygobius cocosensis*, *B. cotticeps* and *B. fuscus* (Gobiidae), are known from Mauritius, but the records from tidepools were made elsewhere.

Table 1 does not include pelagic species and fishes that usually forage in open water or near the water surface (e.g. anchovies, herrings, carangids, barracudas or needlefishes), though they can often be observed visiting intertidal habitats, especially mangrove creeks, during the juvenile stage (Bianchi 1985, Whitehead 1985, Whitehead et al. 1988, Aguilar-Perera and Appeldoorn 2007). Ntiba et al. (1993) recorded several representatives of the above-mentioned families from intertidal mangrove creeks in Kenya, for example, *Herklotsichthys quadrimaculatus* (Rüppell, 1837), *Spratelloides delicatulus* (Bennett, 1832), *Caranx ignobilis* (Forsskål in Niebuhr, 1775), *Gnathanodon speciosus* (Forsskål in Niebuhr, 1775), *Trachinotus baillonii* (Lacepède, 1801), *Trachinotus blochii* (Lacepède, 1801), *Chanos chanos* (Forsskål in Niebuhr, 1775), *Hemirhamphus far* (Forsskål in Niebuhr, 1775), *Lobotes surinamensis* (Bloch, 1790), *Sphyraena barracuda* (Catesby, 1771) and *Sphyraena jello* Cuvier, 1829. All of these species are known to occur in Mauritian waters (Fricke 1999), but so far have not been recorded from intertidal habitats in Mauritius.

### Intertidal permanent residents in examined tidepools

Eight permanent residents were recorded from the 23 tidepools examined in September/October 2018 (Table 2, Suppl. material 2). The species number in the pools increased significantly with the proportion of stones (rock) covering the pool bottom (p = 0.05), reflecting the higher number of species recorded from pools in the vicinity of Albion compared to those at the other examined locations.

**Figure 3.**
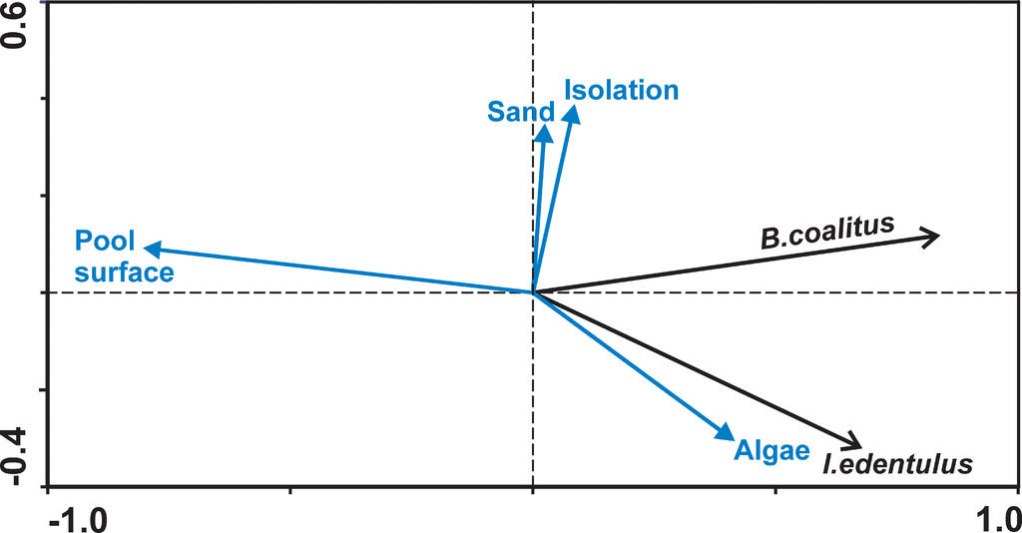
Redundancy analysis (RDA) of the two most common intertidal residents *Bathygobius coalitus* and *Istiblennius edentulus* in the examined tidepools. Axes 1 and 2 explain 66.7% of the variance of species data and 100% that of environmental data. Only environmental parameters with highest explanatory value are shown.

**Figure 4.**
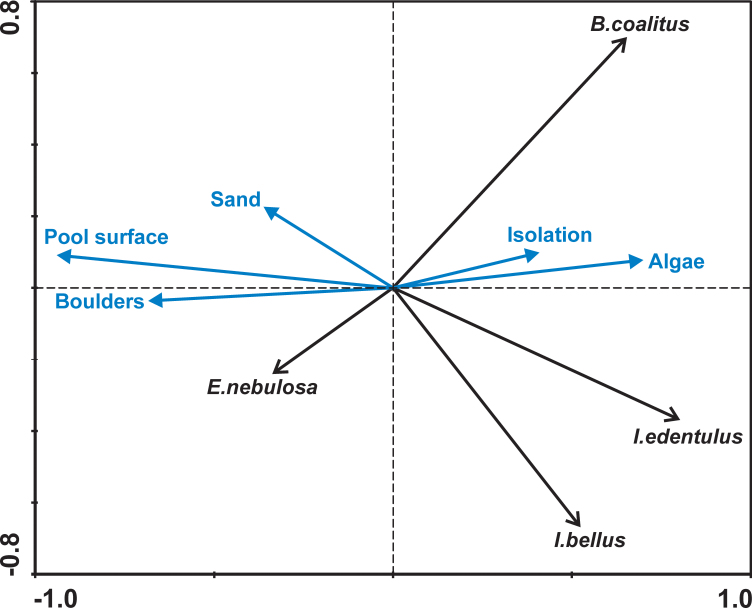
Redundancy analysis (RDA) of the intertidal residents in the examined rockpools at the west coast of Mauritius (AL, LH). Axes 1 and 2 explain 50.0% of the variance of species data and 97.9% that of environmental data. A species fit was set by 15% (meaning that the three species with lowest explanatory value are not shown).

*Bathygobius coalitus* and *Istiblennius edentulus* were the most widespread species in the tidepools. The abundance of *B. coalitus* decreased with increasing depth of the pools (p = 0.04) and with increasing pool surface (p < 0.001). *B. coalitus* occupied very shallow areas of pools, often closely crowded during lowest level of ebb tide. A similar 'overcrowding-effect' was seen in *I. edentulus* with a significantly negative correlation between its abundance and pool surface area during ebb tide (p < 0.001). The influence of these parameters on the occurrence of *B. coalitus* and *I. edentulus* was also confirmed by the RDA. Furthermore, the ordination showed a close relationship between the abundance of *I. edentulus* and the coverage of algae on one side and a relationship between *B. coalitus* and pools with a long period of isolation during the tidal cycle on the other side (Fig. 3). The second RDA calculation, including the rockpools around Albion and Pointe aux Caves, revealed that the surface area and bottom structures of pools, as well as the duration of isolation from in- and out-streaming water during the tidal cycle, may be important parameters that influence the settlement of species (Fig. 4). The starry moray (*Echidna nebulosa*) and the ebony gregory (*Stegastes limbatus*) occur in large pools with a supply of boulders. Species of *Istiblennius* are abundant in pools with high coverage of macro-algae. Whether some of these macro-algae serve as food for these fishes could not be verified. Rather, they were observed feeding on tiny algae, the coverage of which was not estimated because these algae were too small.

## Discussion

Besides the present study, a few further publications specifically dealing with intertidal fishes in the Western Indian Ocean do exist. Durville and Chabanet (2009) found a total of 32 different fish species in intertidal rockpools on the Glorieuses Islands. They denominated 19 of these species as 'typical population' also occurring during the adult stage in these habitats, whereas the remaining 13 species were observed during their juvenile stage only. The examined pools were located high in the infra-littoral zone, thus more or less isolated during long periods of the tidal cycle and they had a surface area of approximately 2 m^2^ during ebb tide. For these reasons, the results by Durville and Chabanet (2009) are comparable with those in our pools. Several taxa, listed as permanent or temporary residents by Durville and Chabanet (2009), were confirmed in Mauritian pools, either by USNM material (collected by Heemstra and co-workers in the year 1995) or through the present study, for example, the temporary residents *Kuhlia mugil*, *Chaetodon lunula*, *Stethojulis albovittata*, *Thalassoma purpureum*, *Acanthurus triostegus*, *Chrysiptera biocellata*, *Chrysiptera glauca*, three species of *Abudefduf* and the permanent residents *Istiblennius dussumieri* and *I. edentulus*. In contrast to the situation in Mauritian pools, Durville and Chabanet (2009) found juveniles and adults of the moray eel *Gymnothorax pictus* and several adults of the eel blenny *Haliophis guttatus* in their small pools. These species met the characters of 'typical' for intertidal pools in the sense of Durville & Chabanet, i.e. the species represented permanent residents in Glorieuses Islands' tidepools. *G. pictus* and *H. guttatus* seem to be rare in Mauritius and no records from tidepools have been published so far. Besides *H. guttatus*, no other Mauritian species of Pseudochromidae is known from tidepools. However, Beckley (1985), Winterbottom (1986) and Burger (1990) confirm that eel blennies may represent permanent intertidal residents and may occur in tidepools in large numbers.

Sindorf et al. (2015)recorded 55 species during an examination of intertidal fishes in Watramu Marine National Park (Kenya), 21 of which were permanent residents. Twenty-five of these species also occur in Mauritian tidepools; however, we assigned several of them to different resident categories (see remarks in Table 1). Five wrasses and seven damselfishes coincided in Kenyan and Mauritian tidepools, but not a single permanent resident of either gobies or blennies was found in both studies(?).

Two tidepool studies took place in the northern Indian Ocean. Ghanbarifardi and Malek (2009) examined pool communities along the Iranian coast (Persian Gulf and Gulf of Oman). The large majority of fish (93.5%) represented permanent residents of gobies and blennies; three species of either family were most abundant. The remaining 6.5% of individuals comprised eight species from six families, all of which are apparently temporary residents. None of the abundant species along the Iranian coast occurs in Mauritius (cf. Table 2 in Ghanbarifardi and Malek 2009). Tsering et al. (2012) published a study from Goa (India). All of the examined pools were small (surface < 1 m^2^), but their depth ranged from 0.1 to 0.7 m. Seven fish species were recorded altogether, including gobies, blennies and *Abudefduf sordidus* which may represent permanent residents, but information referring to this category was not given by the authors. The pools from Goa were dominated by *Istiblennius dussumieri*, the only species that occurs in Mauritius as well.

In the present field study, the total number of permanent intertidal residents was low compared with the results of the field trip by Heemstra and co-workers in the year 1995 (in Fricke 1999), Durville and Chabanet (2009) or Sindorf et al. (2015). This low number of residents could be due to the low depth and small size of most of the examined pools. In particular, Heemstra et al. (in Fricke 1999) recorded a larger number of permanent residents in tidepools in Albion and at the lighthouse in Pointe aux Caves, including two longfins of the genus *Plesiops*, the gobies *Eviota prasina* and *Hetereleotris zonata* and the triplefin *Enneapterygius philippinus*. Some of these pools were re-examined during the present study, but neither the longfins, nor the triplefins or the mentioned genera of gobies were confirmed, even though these species are distinct and not particularly cryptic. It seems likely that the apparent decrease in intertidal species around Albion between 1995 and 2018 has fundamental reasons, for example, environmental change. Eutrophication and industrial wastes including, in particular, metal pollution in the Port Louis area, effects of eutrophication due to a high input of nitrate probably of agricultural origin in the Flic en Flac coastal area and effects due to increasing tourism have been recorded at the Mauritian west coast next to Albion (Ramessur 2013). Seawater pollution had already caused a decline in coverage of live corals by 10-30% in coastal lagoons around Mauritius by 2012 (Ramessur 2013). It is conceivable that pollution affected the diversity of intertidal fish as well. We know that *I. edentulus*, one of the most abundant species in our field study, may tolerate poor water quality (P. Bourjon, verb. commun.).

A comparably low number of resident species does not necessarily mean low abundances of fish. On one hand, a number of opportunists and transients appeared especially in larger pools (cf. species in Table 1). On the other hand, the abundances of some resident species were not at all low. The total abundance of permanent residents averaged 5.31 per m^2^ (SD ± 2.1) in pools ≤ 6 m^2^ and 1.24 (SD ± 1.54) in pools ≥ 10 m^2^, the maximum value being 9 individuals/m^2^. Lundquist and Pinkerton (2008), who examined tidal pools in New Zealand, estimated an abundance of 10 fish/m^2^ across their intertidal study area, Bennett and Griffiths (1984) counted 7.42 individuals per m^2^ in South Africa, while González-Murcia et al. (2016) found mean total abundances of between 5 fish/m^2^ in high shore pools and 12 fish/m^2^ in pools at lower shore sections in El Salvador, but opportunist and transients were included in these studies.

It is not surprising that larger tidepools, containing more algal and rock ledge cover, host a larger and more diverse population of fish. Pool depth, volume and also the variety of microhabitats such as presence of shells, pebbles and rock ledges influence richness and total abundance of fish strongly (Mahon and Mahon 1994, White et al. 2015). White et al. (l.c.) examined very small pools with an area of 20 cm^2^ to 8 m^2^ in New South Wales (Australia) and included all 27 recorded fish species in their analysis. In the present study, we used another approach focusing on permanent residents. The species number increased with pool size as well, but did not yield a significant result due to the much lower number of species in question. However, the results show that specific pool microhabitats are associated with the occurrence and abundance of particular species. After exclusion of fundamental oceanographic parameters, because lagoon or wave-exposed open sea sites influence stone and sand coverage of examined pools differently, facilities like coverage of algae and presence of boulders contributed mainly to the occurrence of certain permanent residents (cf. Fig. 4). The abundance of the two most common species, *B. coalitus* and *I. edentulus*, correlated negatively with the pool surface area (Fig. 3), that of *B. coalitus* also with the depth of the pools. As deep and large pools tend to contain predators (White et al. 2015), shallow and small pools with a long isolation period during the tidal cycle obviously may be beneficial for these permanent residents.

It is well known that a large number of intertidal fishes stay in intertidal habitats only temporarily, most of which use the shelter in these narrow and more or less isolated habitats as juveniles, but move to deeper water once they reach the adult stage. This can be observed in mangrove areas (Nagelkerken et al. 2000, Laegdsgaard and Johnson 2001, Ikejima et al. 2003, Mumby et al. 2004, Jaxion-Harm et al. 2012) and estuaries (Miller et al. 1985, Able 2005, Vasconcelos et al. 2008, Figueiredo and Pessanha 2016), in which vegetated habitats within estuaries tend to harbour higher densities of many fish species than unvegetated substrates (Sogard 1992).

Tidepools also offer shelter for juvenile temporary residents (Mahon and Mahon 1994, Gibson and Yoshiyama 1999, Durville and Chabanet 2009, Ghanbarifardi and Malek 2009, Murase 2013, Sindorf et al. 2015, González-Murcia et al. 2016). In Mauritius, we identified 175 temporary residents (60% of all intertidal species in the study area, cf. Table 1) and in our field study, 16 of those species represented juvenile temporary residents that correspond to 66.7% of recorded species in shallow tidepools. Moray eels (Muraenidae), gobies (Gobiidae), damselfishes (Pomacentridae), groupers (Serranidae) and surgeonfishes (Acanthuridae) comprise temporary residents that can be found most frequently in the tidepools of Mauritius.

## Conclusions

A considerable portion of littoral fishes occurs in the intertidal environment and the present study yielded a large number of intertidal species in Mauritian waters. However, knowledge about local distribution and ecology of these species is still much more fragmentary than that of intertidal species in the North-eastern Pacific or North Atlantic. Intertidal habitats are prone to human influences. The comparison of past and present data from Mauritius suggests a decline of intertidal residents over the last decades. A more detailed knowledge of intertidal communities and more long-term data could enable us to use intertidal fishes as indicators of environmental change and human impact.

For this reason, a future monitoring of tidepool communities and more detailed analyses, for example, with respect to the distribution of feeding types in the communities and the linking of community parameters to parameters of water quality, is highly desirable, not only in Mauritius but also elsewhere.

## Supplementary Material

74cc4b78-ae9b-56ee-b21c-7139a57a44a1 10.3897/BDJ.7.e36754.suppl1 Supplementary material 1 Tidepool characteristics Data type: Ecological data
Brief description: Excel file (*.xlsx) of tidepool parameters. Rows are tidepool parameters and columns are sample sites. Abbreviation of locations: AL – Albion; BB – Blue Bay; LH – Lighthouse at Pointe aux Caves; PE – Péreybère.
Description of tidepool parameters: Ao_ebb - water surface area during ebb tide (in m^2^); Ao_flow - water surface area during flow tide (in m^2^); Depth - pool depth during ebb tide (in cm); Isolation - the duration within one tidal cycle in which water did not stream into or out of the pool (in % of one tidal cycle); S_sand, S_stone, S_algae - bottom coverage with substrates during low tide, i.e. sand, stones including rocks and boulders or macro-algae (in %); Boulders – bottom coverage with boulders during low tide (in %). Note that boulders are structures that provide space for hiding places. Their percentage of bottom coverage is part of 'S_stone'.
Additionally, latitudes and longitudes of the pools are given.
File: oo_321510.xlsx https://binary.pensoft.net/file/321510 Erik Arndt and Ronald Fricke

914c74e9-48ff-593c-aa99-0cce5549c8b1 10.3897/BDJ.7.e36754.suppl2 Supplementary material 2 Recorded permament intertidal resident fish species in shallow tidepools of Mauritius Data type: Ecological data
Brief description: Excel file (*.xlsx) of counted permanent resident species in tidepools. Rows are species and columns are sample sites. Abbreviation of locations: AL – Albion; BB – Blue Bay; LH – Lighthouse at Pointe aux Caves; PE – Péreybère.
Additionally, the sum of species, sum of individuals and two diversity parameters (Brillouin's Index and evenness after Buzas & Gibson) are given.
File: oo_321511.xlsx https://binary.pensoft.net/file/321511 Erik Arndt and Ronald Fricke

## Figures and Tables

**Table 1.** List of intertidal fish species with their residency status and references to their tidepool records in Mauritius. Genus and species classification follows Fricke et al. (2019). Status: O – opportunists, also known as secondary or temporary residents; R - permanent intertidal residents (**R**! - permanent tidepool inhabitants); T - transients or visitors ; ? – current knowledge does not allow the determination of the status with certainty. Sources: EA - E. Arndt, unpubl. obs. 2017/2018; * - tidepool record not in Mauritius but elsewhere.

**Families and species**	**Status**	**Sources and Remarks**	**Museum material from tidepools in Mauritius**
**Acanthuridae Bonaparte, 1835 – Surgeonfishes, unicornfishes**			
*Acanthurus lineatus* (Linnaeus, 1758)	O	Ntiba et al. 1993*, Fricke et al. 2013* Remark: No detailed locality published from Mauritius.	
*Acanthurus nigrofuscus* (Forsskål in Niebuhr, 1775)	O	Fricke 1999	USNM 348969 (1)
*Acanthurus polyzona* (Bleeker, 1868)	O	Fricke 1999, Fricke et al. 2009*	USNM 341559 (1)
*Acanthurus triostegus* (Linnaeus, 1758)	O	Beckley 1985*, Durville and Chabanet 2009*, Fricke et al. 2009*, Fricke et al. 2013*, EA 2018 Remarks: O acc. to Durville and Chabanet (2009) and Cox et al. (2011); R acc. to Sindorf et al. (2015).	USNM 344298 (65), 348973 (16), 348975 (24)
*Acanthurus xanthopterus* Valenciennes, 1835	O	Ntiba et al. 1993*, SMNS material* (New Caledonia) Remark: Records from Mauritius possibly subtidal (Fricke 1999).	
*Ctenochaetus striatus* (Quoy & Gaimard, 1825)	O	Fricke 1999	USNM 348986 (2)
*Naso brevirostris* (Cuvier, 1829)	O	Ntiba et al. 1993* Remarks: In mangrove creeks (Ntiba et al. 1993). Records from Mauritius possibly subtidal (Fricke 1999).	
*Naso unicornis* (Forsskål in Niebuhr, 1775)	O	Fricke 1999, Fricke et al. 2013*	USNM 349093 (1), 349094 (1)
**Ambassidae Klunzinger, 1870 – Asiatic glassfishes**			
*Ambassis natalensis* (Lacepède, 1802)	T	Fricke 1999 (under *A. ambassis*), Fricke et al. 2009* Remarks: Freshwater, estuarine and marine. From mouth of a stream with mangrove shore in Mauritius.	BMNH 2002.6.30.1197-1296
**Anguillidae Rafinesque, 1810 – Freshwater eels**			
*Anguilla bengalensis* Gray, 1831	T	Fricke 1999, Keith et al. 1999* Remark: Freshwater, estuarine and marine.	
*Anguilla bicolor* McClelland, 1841	T	Fricke 1999, Keith et al. 1999* Remark: Freshwater, estuarine and marine.	
*Anguilla marmorata* Quoy & Gaimard, 1824	T	Fricke 1999, Keith et al. 1999* Remark: Freshwater, estuarine and marine.	
*Anguilla mossambica* (Peters, 1852)	T	Fricke 1999, Keith et al. 1999* Remark: Freshwater, estuarine and marine.	
**Antennariidae Jarocki, 1822 – Frogfishes**			
*Antennarius commerson* (Lacepède, 1798)	O	Fricke et al. 2009*	
*Antennarius hispidus* (Bloch & Schneider, 1801)	O	Fricke 1999* Remarks: No detailed locality published from Mauritius. Intertidal record from Réunion.	
*Antennarius striatus* (Shaw, 1794)	O	Pietsch 1986* Remark: No detailed locality published from Mauritius (Fricke 1999).	
*Antennatus coccineus* (Lesson, 1831)	O	Fricke et al. 2009*	
*Antennatus nummifer* (Cuvier, 1817)	O	Fricke et al. 2009*	
*Antennatus tuberosus* (Cuvier, 1817)	O	Fricke 1999 Remark: Mauritian tidepool records Heemstra et al. 1995 (USNM material).	USNM 349867 (1), 349868 (1)
*Histrio histrio* (Linnaeus, 1758)	O	Roux 2013* Remark: No detailed locality published from Mauritius (Fricke 1999).	
**Apogonidae Günther, 1859 – Cardinal fishes**			
*Apogon caudicinctus* Randall & Smith, 1988	O	Remark: USNM material from tidepools (0-1 m) in Mauritius.	USNM 349769 (2)
*Apogon semiornatus* Peters, 1876	O	Fricke et al. 2013*	
*Apogonichthys ocellatus* (Weber, 1913)	O	Fricke 1999* Remark: Records from Mauritius possibly subtidal. Intertidal record from Réunion.	
*Fowleria variegata* (Valenciennes, 1832)	O	Fricke 1999 (SMNS material, Australia)	USNM 346958 (1)
*Ostorhinchus aureus* (Lacepède, 1802)	O	Fricke 1999* Remarks: Records from Mauritius possibly subtidal. Intertidal record from Réunion.	
*Ostorhinchus holotaenia* (Regan, 1905)	O	Fricke et al. 2013*	
*Ostorhinchus taeniophorus* (Regan, 1908)	O	Fricke 1999, Fricke et al. 2009*, Fricke et al. 2013*; EA 2018 Remark: R acc. to Sindorf et al. (2015)	USNM 349764 (1), 349766 (17)
*Pristiapogon fraenatus* (Valenciennes, 1832)	O	Ntiba et al. 1993*, Fricke 1999* Remarks: Records from Mauritius possibly subtidal. Intertidal record from Réunion.	
*Rhabdamia gracilis* (Bleeker, 1856)	O	Fricke et al. 2013* Remark: Only records from deeper water known in Mauritius (Fricke 1999).	
*Siphamia mossambica* Smith, 1955	O	Fricke et al. 2009* Remark: No detailed locality published from Mauritius (Fricke 1999).	
**Aulostomidae Rafinesque, 1815 – Trumpetfishes**			
*Aulostomus chinensis* (Linnaeus, 1766)	T	Fricke 1999, EA 2018	
**Balistidae Rafinesque, 1810 – Triggerfishes**			
*Balistoides viridescens* (Bloch & Schneider, 1801)	O	Fricke et al. 2009*, Fricke et al. 2013*	
*Rhinecanthus aculeatus* (Linnaeus, 1758)	O	Fricke et al. 2009*, Fricke et al. 2013*, EA 2018 Remark: R acc. to Sindorf et al. (2015).	
**Blenniidae Rafinesque, 1810 – Blennies**			
*Alloblennius anuchalis* Springer & Spreitzer, 1978	**R**!	Springer and Spreitzer 1978, Fricke 1999	
*Alloblennius parvus* Springer & Spreitzer, 1978	**R**!	Springer and Spreitzer 1978*, Fricke 1999 Remark: In the surge zone of rocky coasts, over edges of drop-offs and in tidepools (Springer and Spreitzer 1978).	
*Alticus monochrus* Bleeker, 1869	R	Bhikajee 1996, Fricke 1999, Fricke et al. 2009*, Fricke et al. 2013*, EA 2017, 2018 Remarks: On rocks in the surge zone and outside water, occasionally in tidepools, 0-1m (Bhikajee 1996, USNM material). Temporarily in tidepools, e.g. resting during day and at night (Arndt, unpubl. obs., Bhikajee and Green 2002, Bhikajee et al. 2006).	SMNS 16884 (1); USNM 344364 (40), 344265 (1), 344266 (2)
*Antennablennius bifilum* (Günther, 1861)	**R**!	Fricke 1999, Fricke et al. 2009*, Fricke et al. 2013*, Roux 2013* Remark: From the surge zone to 5 m, also in tidepools.	SMNS 16908 (1); USNM 341904 (1)
*Aspidontus tractus* Fowler, 1903	O	Fricke 1999 (as *A. taeniatus tractus*) Remarks: Only records from deeper water known in Mauritius. Intertidal record from Réunion.	
*Blenniella chrysospilos* (Bleeker, 1857)	R	Fricke 1999, Fricke et al. 2013*	SMNS 16943 (2)
*Blenniella gibbifrons* (Quoy & Gaimard, 1824)	**R**!	Fricke 1999, Cox et al. 2011*, Fricke et al. 2013* Remark: R acc. to Cox et al. (2011).	SMNS 16883 (21), 16929 (2); USNM 344269 (1)
*Blenniella periophthalmus* (Valenciennes, 1836)	**R**!	Fricke 1999, Fricke et al. 2013*, EA 2017, 2018	SMNS 16894 (1), 16904 (2); USNM 344270 (1), 344271 (14)
*Cirripectes castaneus* (Valenciennes, 1836)	O	Fricke 1999	
*Cirripectes quagga* (Fowler & Ball, 1924)	O	Fricke 1999	
*Cirripectes randalli* Williams, 1988	?O/R	Fricke 1999	USNM 344250 (3)
*Cirripectes stigmaticus* Strasburg & Schultz, 1953	O	Fricke 1999* Remarks: Material from Mauritius includes subtidal records only. Intertidal record from Réunion.	
*Cirrisalarias bunares* Springer, 1976	**R**!	Fricke 1999 Remark: Adults in tidepools, surge channels and outer reef slopes near surface (Allen and Erdmann 2012).	
*Dodekablennos fraseri* Springer & Spreitzer, 1978	**R**!	Fricke 1999, Fricke et al. 2009*	USNM 343646 (1), 344272 (1), 344273 (7)
*Enchelyurus kraussii* (Klunzinger, 1871)	?O/R	Fricke et al. 2009* Remark: Material from Mauritius includes subtidal records only (Fricke 1999).	
*Entomacrodus lemuria* Springer & Fricke, 2000	R	Fricke 1999 (as *Entomacrodus* sp.), Springer and Fricke 2000, Fricke et al. 2009* Remark: In the near shore surge zone (Springer and Fricke 2000) and in tidepools (leg. Heemstra et al. 1995, in USNM material).	USNM 341905, 339747 (4 paratypes)
*Entomacrodus epalzeocheilos* (Bleeker, 1859)	R	Fricke 1999, Fricke et al. 2009* Remark: In intertidal areas, e.g. reef flats exposed to waves, rocky shores and tidepools (Allen and Erdmann 2012).	USNM 344254 (1)
*Entomacrodus striatus* (Valenciennes, 1836)	**R**!	Fricke 1999, Fricke et al. 2009*, EA 2017 Remark: "Accidental visitor" (= T) acc. to Murase (2015).	USNM 341906 (11), 344255 (3)
*Entomacrodus vermiculatus* (Valenciennes, 1836)	**R**!	Fricke 1999 Remark: Adults are found in the intertidal area, actively shuttling back and forth between rockpools and air (Martin and Bridges 1999).	USNM 344256 (6)
*Istiblennius bellus* (Günther, 1861)	**R**!	Fricke 1999, EA 2017, 2018 Remark: Adults in intertidal flats and rock shores (Allen and Erdmann 2012).	SMNS 16877 (43), 16900 (4); USNM 344258 (4), 344259 (12), 344260 (22)
*Istiblennius dussumieri* (Valenciennes, 1836)	**R**!	Fricke 1999, Durville and Chabanet 2009*, EA 2018 Remarks: Rocky shorelines and mangrove areas (Springer and Williams 1994). R acc. to Durville and Chabanet (2009) and Murase (2015).	SMNS 16923 (2)
*Istiblennius edentulus* (Forster & Schneider, 1801)	**R**!	[Bibr B5239440][Bibr B5298309]*, Durville and Chabanet 2009*, Fricke et al. 2009*, Fricke et al. 2013*, EA 2017, 2018 Remark: Intertidal, may remain out of water under rocks or seaweeds (Martin and Bridges 1999, Kuiter and Tonozuka 2001).	SMNS 16875 (12), 16907 (5), 16935 (2); USNM 342078 (4), 344261 (40), 344262 (20), 344263 (141)
*Istiblennius spilotus* Springer & Williams, 1994	**R**!	Fricke 1999, Durville and Chabanet 2009*, Fricke et al. 2009*	SMNS 16882 (1)
*Istiblennius steindachneri* (Pfeffer, 1893)	R	Fricke 1999* Remarks: Material from Mauritius includes subtidal records only. Intertidal record from Réunion.	
*Mimoblennius rusi* Springer & Spreitzer, 1978	**R**!	Fricke 1999 Remarks: In tidepools and rocky surge areas (Allen and Erdmann 2012). Most records from Mauritius much deeper.	
*Omobranchus elongatus* (Peters, 1855)	R	Fricke 1999 Remarks: In rocky reefs with oysters and in estuaries (Allen and Erdmann 2012). Mauritian record from a mangrove area (leg. Heemstra et al. 1995, USNM material).	SMNS 16919 (1); USNM 341865 (3)
*Parenchelyurus hepburni* (Snyder, 1908)	R	Fricke 1999 Remark: In the intertidal zone (Masuda et al. 1984).	
*Salarias fasciatus* (Bloch, 1786)	R	Fricke 1999 Remarks: Intertidal (Kuiter and Tonozuka 2001), R acc. to Sindorf et al. (2015). No exact locality published for Mauritius.	
**Bothidae Smitt, 1892 – Left eye flounders**			
*Bothus mancus* (Broussonet, 1782)	O	Ntiba et al. 1993*, Durville and Chabanet 2009* Remark: Records from Mauritius possibly subtidal (Fricke 1999).	
*Bothus pantherinus* (Rüppell, 1830)	O	Ntiba et al. 1993* Remark: Records from Mauritius possibly subtidal (Fricke 1999).	
**Callionymidae Bonaparte, 1831 – Dragonets**			
*Diplogrammus infulatus* Smith, 1963	**R**!	Fricke et al. 2009*, Fricke et al. 2013*, EA 2017 Remark: In tidepools with seaweeds and shallow waters (Fricke 1986).	
**Chaetodontidae Rafinesque, 1815 – Butterflyfishes**			
*Chaetodon auriga* Forsskål in Niebuhr, 1775	O	Fricke 1999* Remarks: Records from Mauritius possibly subtidal. Intertidal record from Réunion.	
*Chaetodon lunula* (Lacepède, 1802)	T	Fricke 1999, Durville and Chabanet 2009*, Fricke et al. 2009*, Fricke et al. 2013*, Roux 2013* Remarks: R acc. to Sindorf et al. (2015); T acc. to Cox et al. (2011) and acc. to Murase (2015).	USNM 348121 (4), 348122 (7), 348123 (9)
*Chaetodon vagabundus* Linnaeus, 1758	?T/O	Fricke 1999* Remarks: Records from Mauritius possibly subtidal. Intertidal record from Réunion.	
*Heniochus acuminatus* (Linnaeus, 1758)	?T/O	Ntiba et al. 1993* Remarks: Kenyan intertidal record from mangrove creeks. No detailed locality published from Mauritius (Fricke 1999).	
**Clinidae Swainson, 1839 – Klipfishes, kelp blennies**			
*Springeratus polyporatus* Fraser, 1972	**R**!	Fricke 1999, Fricke et al. 2009* Remark: All clinids are R acc. to Griffiths (2003b).	SMNS 16895 (1), 16913 (1)
**Congridae Kaup, 1856 – Conger eels**			
*Conger cinereus* Rüppell, 1871	O	Fricke 1999, Fricke et al. 2009*, Fricke et al. 2013*	SMNS 16888 (2); USNM 342780 (2), 342781 (1), 342785 (9)
*Conger wilsoni* (Bloch & Schneider, 1801)	O	Fricke et al. 2009* Remark: So far only known from Rodrigues (Fricke et al. 2009).	
**Creediidae Waite, 1899 – Sand burrowers**			
*Chalixodytes tauensis* Schultz, 1943	O	Fricke et al. 2009* Remark: Records from Mauritius possibly subtidal (Fricke 1999 as *C. chamaeleontoculis*).	
*Limnichthys nitidus* Smith, 1958	O	Fricke et al. 2009* Remark: Records from Mauritius possibly subtidal (Fricke 1999).	SMNS 16887 (1)
**Dactylopteridae Gill, 1861 – Flying gurnards**			
*Dactyloptena orientalis* (Cuvier, 1829)	?O/T	Ntiba et al. 1993* Remark: Records from Mauritius possibly subtidal (Fricke 1999).	
**Dinematichthyidae Whitley, 1928 – Viviparous brotulas**			
*Mascarenichthys heemstrai* Schwarzhans & Møller, 2007	O	Fricke 1999, Schwarzhans and Møller 2007, Fricke et al. 2009*	USNM 349826 (1)
**Diodontidae Billberg, 1833 - Boxfishes**			
*Diodon hystrix* Linnaeus, 1758	?O/T	Ntiba et al. 1993* Remark: No detailed locality published from Mauritius (Fricke 1999).	
**Eleotridae Bonaparte, 1835 – Sleepers**			
*Eleotris fusca* (Bloch & Schneider, 1801)	R	Fricke 1999, Keith et al. 1999* Remark: In Mauritius found in coastal creeks and mangrove areas.	
*Eleotris mauritiana* Bennett, 1832	R	Fricke 1999, Keith et al. 1999* Remark: In coastal creeks and streams with mangrove areas of Mauritius.	USNM 347883 (14)
*Hypseleotris cyprinoides* (Valenciennes, 1837)	?R/O	Keith et al. 1999* Remarks: Inhabiting freshwater streams, also entering estuaries (Froese and Pauly 2018). No detailed locality published from Mauritius (Fricke 1999).	
*Ophiocara porocephala* (Valenciennes, 1837)	?R/O	Fricke et al. 2009* Remarks: In estuaries, river mouths and freshwater creeks, upstream from the tidal zone (Allen 1991, Rainboth 1996). No detailed locality published from Mauritius (Fricke 1999).	
**Fistulariidae Stark, 1828 – Flutemouths**			
*Fistularia commersonii* Rüppell, 1838	T	Fricke 1999, EA 2018	USNM 348086 (1)
**Gerreidae Bleeker, 1859 – Mojarras**			
*Gerres filamentosus* Cuvier, 1829	O	Ntiba et al. 1993*, Fricke et al. 2009* Remarks: Found in marine, brackish and freshwater; juveniles in mangrove areas and tidal creeks (Allen 1991, Allen et al. 2002). No detailed locality published from Mauritius (Fricke 1999).	
*Gerres oyena* (Forsskål in Niebuhr, 1775)	O	Ntiba et al. 1993*, Fricke et al. 2013* Remark: Mauritian record in mouth of a stream with mangrove shore, 0-1m.	USNM 349511 (19)
**Gobiidae Cuvier, 1816 – Gobies**			
*Amblygobius albimaculatus* Rüppell, 1830	O	Maugé 1986*, Ntiba et al. 1993* Remarks: In estuaries and mangrove areas. No detailed locality published from Mauritius (Fricke 1999).	
*Asterropteryx semipunctata* Rüppell, 1830	O	Fricke 1999, Fricke et al. 2013* Remark: T acc. to Murase (2015).	SMNS 16945 (1)
*Awaous commersoni* (Schneider in Bloch & Schneider, 1801)	O	Fricke et al. 2013* Remark: Records from Mauritius possibly subtidal (Fricke 1999).	
*Bathygobius coalitus* (Bennett, 1832)	**R**!	Fricke 1999, Fricke et al. 2009*, Cox et al. 2011*, Murase 2015*, EA 2017, 2018 Remark: USNM material (leg. Heemstra et al. 1995) from tidal rockpools and a mangrove area.	USNM 348023 (26), 348024 (72), 348025 (44), 348026 (4)
*Bathygobius cocosensis* (Bleeker, 1854)	**R**!	Fricke 1999, Griffiths 2003b*, Fricke et al. 2009*, Cox et al. 2011*, Roux 2013*, Murase 2015*	Fricke et al. 2009SMNS 16917 (1), 16930 (1)
*Bathygobius cotticeps* (Steindachner, 1879)	**R**!	Fricke et al. 2009*, Cox et al. 2011*, Fricke et al. 2013* Remark: Only known from Rodrigues (Fricke 1999).	
*Bathygobius fuscus* (Rüppell, 1830)	**R**!	Fricke 1999, Fricke et al. 2009*, Fricke et al. 2013*	SMNS 16886 (2), 16909 (3), 16928 (1)
*Callogobius flavobrunneus* (Smith, 1958)	**R**!	Fricke 1999 Note: *Callogobius* sp listed as R by Griffiths (2003b).	USNM 348030 (11)
*Cotylopus acutipinnis* Guichenot, 1863	O	Maugé 1986*, Fricke 1999 (as *Cotylopus* sp.), Keith et al. 1999*, Fricke et al. 2009*	
*Eviota distigma* Jordan & Seale, 1906	?R/O	SMNS material* (Australia) Remark: Records from Mauritius possibly subtidal (Fricke 1999).	
*Eviota nigripinna* Lachner & Karnella, 1980	?R/O	Fricke 1999 Remarks: Records from Mauritius possibly subtidal. Intertidal record from Réunion.	
*Eviota prasina* (Klunzinger, 1871)	**R**!	Fricke 1999, Fricke et al. 2009*, Fricke et al. 2013*	SMNS 16916 (4), 16936 (4); USNM 347920 (9), 347921 (3), 347927 (9)
*Favonigobius reichei* (Bleeker, 1854)	R	Fricke 1999 Remarks: In weedy areas of intertidal zone, also in mangroves and estuaries. Mauritian record in mouth of a stream with mangroves.	USNM 347776 (4)
*Fusigobius maximus* (Randall, 2001)	?R/O	Fricke 1999 (as *Coryphopterus neophytus*), Kuiter and Tonozuka 2001*, Fricke et al. 2009* Remarks: In tidal reef flats and shallow lagoons. Only deeper records known from Mauritius (Fricke 1999).	SMNS 16944 (2)
*Glossogobius giuris* (Hamilton, 1822)	T	Keith et al. 1999* Remarks: No detailed published record from Mauritius (Fricke 1999).	
*Gnatholepis anjerensis* (Bleeker, 1851)	O	Fricke 1999, Cox et al. 2011*, EA 2017, 2018 Remarks: R acc. to Cox et al. (2011) and Sindorf et al. (2015). Only juveniles in Mauritian tidepools (Arndt, unpubl. obs.).	
*Gnatholepis cauerensis* (Bleeker, 1853)	O	Fricke 1999 Remark: R acc. to Sindorf et al. (2015).	
*Gobiodon rivulatus* (Rüppell, 1830)	O	Maugé 1986* Remarks: No detailed published record from Mauritius (Fricke 1999); one subtidal record in USNM material.	USNM 400202 (2)
*Hetereleotris apora* (Hoese & Winterbottom, 1979)	?O/T	Fricke 1999 Remarks: Only subtidal records known from Mauritius. Intertidal record from Réunion.	
*Hetereleotris georgegilli* Gill, 1998	O	Gill 1998, Fricke 1999, Fricke et al. 2009*	
*Hetereleotris poecila* (Fowler, 1946)	O	Fricke 1999 Remark: USNM material (leg. Heemstra et al. 1995) from tidal rockpools.	USNM 344333 (5)
*Hetereleotris vinsoni* Hoese, 1986	?O/R	Fricke 1999	
*Hetereleotris zanzibarensis* (Smith, 1958)	O	Fricke 1999, Fricke et al. 2009*	USNM 344325 (2)
*Hetereleotris zonata* (Fowler, 1934)	**R**!	Fricke 1999, Randall 1995*	USNM 344334 (5)
*Istigobius decoratus* (Herre, 1927)	O	Fricke 1999, EA 2017, 2018 Remarks: Only juveniles in Mauritian tidepools (Arndt, unpubl. obs.); SMNS material, Taiwan.	USNM 347858 (2), 347860 (1)
*Oxyurichthys lonchotus* (Jenkins, 1903)	T	Fricke 1999 Remark: USNM material from a mangrove area.	USNM 347778 (1)
*Periophthalmus kalolo* Lesson, 1831	R	Fricke 1999 Remarks: No detailed locality published from Mauritius. Active and hunting at low tide in the intertidal zone (Martin and Bridges 1999).	
*Priolepis cincta* (Regan, 1908)	O	Fricke 1999, Fricke et al. 2013*, Roux 2013*	USNM 344220 (1)
*Priolepis semidoliata* (Valenciennes, 1837)	?O/R	Fricke 1999 Remark: R acc. to Murase (2015).	SMNS 16891 (2); USNM 344222 (1), 344223 (3), 344232 (10)
*Sicyopterus lagocephalus* (Pallas, 1770)	O	Maugé 1986*, Fricke 1999, Keith et al. 1999* Remark: Only freshwater records are known from Mauritius.	
*Stenogobius polyzona* (Bleeker, 1867)	T	Keith et al. 1999* Remark: Records from Mauritius possibly subtidal (Fricke 1999).	
*Valenciennea sexguttata* (Valenciennes, 1837)	O	Fricke 1999, EA 2017 Remark: USNM material from tidal rockpools.	USNM 347889 (2)
**Haemulidae Gill, 1885 – Sweetlips, grunts**			
*Diagramma picta* (Thunberg, 1792)	O	Ntiba et al. 1993*; Fricke et al. 2009* Remarks: Kenyan intertidal record in a mangrove creek; also in estuaries and seagrass meadows (Kuiter and Tonozuka 2001). No detailed locality published from Mauritius (Fricke 1999).	
*Plectorhinchus gaterinus* (Forsskål in Niebuhr, 1775)	O	Ntiba et al. 1993* Remarks: Found in mangrove creeks, near estuaries (Bianchi 1985). No detailed locality published from Mauritius (Fricke 1999).	
*Plectorhinchus gibbosus* (Lacepède, 1802)	O	Fricke 1999 Remark: USNM material from a mangrove area.	USNM 349308 (1)
**Holocentridae Bonaparte, 1833 – Squirrelfishes, soldierfishes**			
*Myripristis seychellensis* Cuvier, 1829	O	Fricke 1999	USNM 349254 (1)
*Neoniphon sammara* (Forsskål in Niebuhr, 1775)	O	Fricke 1999	USNM 348936 (1)
*Sargocentron diadema* (Lacepède, 1802)	O	Fricke et al. 2009* Remark: Records in the lagoon of Mauritius are below the low tide line (Fricke 1999).	
*Sargocentron punctatissimum* (Cuvier, 1829)	O	Fricke 1999, Fricke et al. 2009* Remark: *Sargocentron* sp was listed as O by González-Murcia et al. (2016).	SMNS 16924 (1); USNM 348945 (9), 348946 (6), 348949 (6)
**Kuhliidae Jordan & Evermann, 1896 – Flagtails**			
*Kuhlia caudavittata* (Lacepède, 1802)	O	Fricke 1999, EA 2017, 2018	SMNS 16878 (45)
*Kuhlia mugil* (Forster in Bloch & Schneider, 1801)	O	Fricke 1999, Durville and Chabanet 2009*, Fricke et al. 2013*, Roux 2013*, EA 2018 Remarks: R acc. to Sindorf et al. (2015), but T acc. to Griffiths (2003b) and Murase (2015).	SMNS 16879 (11); USNM 348951 (31), 348952 (3), 348954 (3), 349508 (9), 349509 (12), 349510 (2)
*Kuhlia rupestris* (Lacepède, 1802)	O	Fricke et al. 2009* Remark: Records from Mauritius possibly subtidal (Fricke 1999).	
**Labridae Cuvier, 1816 – Wrasses**			
*Anampses meleagrides* Valenciennes, 1840	T	EA 2018	
*Cheilinus chlorourus* (Bloch, 1791)	T	Fricke et al. 2013* Remark: Records from Mauritius possibly subtidal (Fricke 1999).	
*Cheilinus oxycephalus* Bleeker, 1853	T	Fricke 1999	USNM 348611 (1)
*Cheilio inermis* (Forsskål in Niebuhr, 1775)	T	Ntiba et al. 1993* Remarks: Kenyan intertidal record in a mangrove creek; records from Mauritius subtidal (Fricke 1999).	
*Coris aygula* Lacepède, 1801	T	Fricke et al. 2009* Remarks: No detailed locality published from Mauritius (Fricke 1999).	
*Coris cuvieri* (Bennett, 1831)	T	Fricke et al. 2009* Remarks: No detailed locality published from Mauritius (Fricke 1999).	
*Coris formosa* (Bennett, 1830)	T	Fricke et al. 2009* (as *C. frerei*) Remarks: No detailed locality published from Mauritius (Fricke 1999).	
*Gomphosus caeruleus* Lacepède, 1801	T	Fricke et al. 2013* Remark: Records from Mauritius possibly subtidal (Fricke 1999).	
*Halichoeres hortulanus* (Lacepède, 1801)	T	Fricke et al. 2013*, Sindorf et al. 2015* Remarks: T acc. to Sindorf et al. (2015). Records from Mauritius possibly subtidal (Fricke 1999).	
*Halichoeres lamarii* (Valenciennes, 1839)	T	Fricke 1999 (as *H. marginatus*) Remarks: T acc. to Murase (2015); González-Murcia et al. (2016) listed species of *Halichoeres* as O or T.	SMNS 16925 (3); USNM 348647 (2), 348648 (1)
*Halichoeres nebulosus* (Valenciennes, 1839)	T	Fricke et al. 2013* Remark: T acc. to Murase (2015).	SMNS 16893 (1)
*Halichoeres scapularis* (Bennett, 1832)	T	Fricke et al. 2013*, Sindorf et al. 2015* Remark: Records from Mauritius possibly subtidal (Fricke 1999).	
*Labroides dimidiatus* (Valenciennes, 1839)	T	Fricke 1999, Sindorf et al. 2015*	USNM 348794 (1)
*Stethojulis albovittata* (Bonnaterre, 1788)	T	Fricke 1999, Durville and Chabanet 2009*, Fricke et al. 2013* Remarks: O acc. to Durville and Chabanet (2009); *Stethojulis sp* was listed as T by Cox et al. (2011).	SMNS 16920 (2); USNM 348800 (4), 348801 (2), 348804 (6)
*Stethojulis strigiventer* (Bennett, 1832)	T	Ntiba et al. 1993* Remarks: Kenyan intertidal record from mangrove creek; records from Mauritius subtidal (Fricke 1999).	
*Thalassoma amblycephalum* (Bleeker, 1856)	T	Fricke 1999, Fricke et al. 2013* Remarks: R acc. to Sindorf et al. (2015), but T acc. to Murase 2015.	SMNS 16889 (3), 16918 (2), 16934 (9); USNM 348814 (1), 348820 (2)
*Thalassoma genivittatum* (Valenciennes, 1839)	T	Fricke 1999	SMNS 16933 (1); USNM 348821 (2)
*Thalassoma hardwicke* (Bennett, 1830)	T	Fricke 1999, Fricke et al. 2013*	SMNS 16896 (1); USNM 348812 (1)
*Thalassoma purpureum* (Forsskål in Niebuhr, 1775)	T	Fricke 1999, Durville and Chabanet 2009* Remarks: O acc. to Durville and Chabanet (2009); T acc. to Cox et al. (2011).	USNM 348929 (3), 348930 (1), 348931 (2)
*Thalassoma quinquevittatum* (Lay & Bennett, 1839)	T	Fricke et al. 2009 (as *T. hebraicum*), Fricke et al. 2013* Remark: No detailed locality published from Mauritius.	
*Thalassoma trilobatum* (Lacepède, 1801)	T	Fricke 1999, Fricke et al. 2013*	SMNS 16876 (22), 16903 (10), 16932 (2); USNM 348806 (13), 348807 (7), 348811 (21), 348925 (9), 348926 (4), 348927 (2)
**Leiognathidae Gill, 1893 – Ponyfishes**			
*Leiognathus equulus* (Forsskål in Niebuhr, 1775)	O	Allen 1991*, Ntiba et al. 1993*, Fricke 1999, Kuiter and Tonozuka 2001*, Allen et al. 2002* Remarks: Juveniles common in mangrove areas, estuaries, tidal creeks and sometimes in lower reaches of freshwater streams. USNM material from mouth of a stream with mangrove shore.	USNM 349512 (93)
**Lethrinidae Bonaparte, 1831 – Emperors**			
*Lethrinus harak* (Forsskål in Niebuhr, 1775)	?O/T	Ntiba et al. 1993* Remark: No detailed locality published from Mauritius (Fricke 1999).	
*Lethrinus nebulosus* (Forsskål in Niebuhr, 1775)	?O/T	Ntiba et al. 1993*, Fricke et al. 2009*, SMNS material* (Australia) Remark: Records from Mauritius possibly subtidal (Fricke 1999).	
*Lethrinus lentjan* (Lacepède, 1803)	?O/T	Ntiba et al. 1993* Remarks: No detailed locality published from Mauritius (Fricke 1999).	
**Lutjanidae Gill, 1861 – Snappers**			
*Lutjanus argentimaculatus* (Forsskål in Niebuhr, 1775)	O	Ntiba et al. 1993*, Fricke et al. 2009* Remark: Juveniles and young adults occur in estuaries, lower reaches of freshwater streams and tidal creeks (Fricke et al. 2009).	USNM 349314 (2)
*Lutjanus bohar* (Forsskål in Niebuhr, 1775)	O	Ntiba et al. 1993* Remark: No detailed locality published from Mauritius (Fricke 1999).	
*Lutjanus fulviflamma* (Forsskål in Niebuhr, 1775)	O	Fricke 1999, Ntiba et al. 1993*, SMNS material* (Australia) Remark: USNM material from mangrove area.	USNM 349313 (1)
*Lutjanus fulvus* (Forster, 1801)	O	Fricke 1999 Remark: USNM material from mangrove area.	USNM 349312 (2)
*Lutjanus johnii* (Bloch, 1792)	O	SMNS material* (Taiwan) Remark: No detailed locality published from Mauritius (Fricke 1999).	
*Lutjanus russellii* (Bleeker, 1849)	O	Ntiba et al. 1993*, SMNS material* (Australia) Remark: Only one record (Port Louis, harbour) known from Mauritius (Fricke 1999).	
**Monacanthidae Nardo, 1843 – Filefishes**			
*Aluteres scriptus* (Osbeck, 1765)	O	Ntiba et al. 1993* Remarks: Juveniles found in mangrove creeks in Kenya. Only subtidal records known from Mauritius (EA 2017).	
**Monodactylidae Jordan & Evermann, 1898 – Moonfishes**			
*Monodactylus argenteus* (Linnaeus, 1758)	T	Fricke et al. 2009* Remarks: In bays, estuaries, tidal creeks etc. (Fricke et al. 2009). USNM material from a mangrove area.	USNM 349505 (20)
*Monodactylus falciformis* Lacepède, 1801	T	Ntiba et al. 1993*, Fricke et al. 2009* Remarks: In bays, estuaries, tidal creeks etc. No detailed locality published from Mauritius (Fricke 1999).	
**Moringuidae Gill, 1885 – Spaghetti eels**			
*Moringua ferruginea* Bliss, 1883	?O/T	Fricke 1999	SMNS 16890 (1), 16898 (19), 16922 (10)
*Moringua javanica* (Kaup, 1856)	?O/T	Fricke et al. 2013* Remark: In the Mascarene archipelago, so far only known from Réunion and Rodrigues (Fricke 1999).	
**Mugilidae Jarocki, 1822 – Mullets**			
*Agonostomus telfairii* Bennett, 1832	T	Thomson 1984*, Keith et al. 1999* Remarks: Freshwater, estuarine and marine. No detailed locality published from Mauritius (Fricke 1999).	
*Chelon melinopterus* (Valenciennes, 1836)	T	Fricke 1999 (as *Liza melinoptera*), Fricke et al. 2009*	SMNS 16912 (1)
*Crenimugil crenilabis* (Forsskål in Niebuhr, 1775)	T	Fricke 1999, Harrison and Senou 1999*, Fricke et al. 2009* Remarks: No detailed locality published from Mauritius (Fricke 1999).	
*Crenimugil seheli* (Forsskål in Niebuhr, 1775)	T	Fricke 1999 (as *Moolgarda seheli*), Ntiba et al. 1993* and Keith et al. 1999* (as *Valamugil seheli*) Remarks: Mangrove areas, estuaries, tidepools, also in freshwater (Harrison and Senou 1999).	
*Mugil cephalus* Linnaeus, 1758	T	Fricke 1999, Keith et al. 1999* Remarks: USNM material from a mangrove area. Also in estuaries and rivers (Harrison and Senou 1999).	USNM 349828 (4)
*Osteomugil robustus* (Günther, 1861)	T	Fricke 1999 (as *Valamugil robustus*), Keith et al. 1999* Remark: Freshwater, estuarine and marine.	
*Planiliza macrolepis* (Smith, 1846)	T	Fricke et al. 2013* (as *Chelon macrolepis*) Remark: In the Mascarene archipelago so far only known from Rodrigues (Fricke 1999 as *Liza macrolepis*).	
**Mullidae Rafinesque, 1815 – Goatfishes**			
*Mulloidichthys flavolineatus* (Lacepède, 1801)	O	Fricke 1999 Remark: USNM material from a mangrove area.	USNM 349318 (5)
*Mulloidichthys vanicolensis* (Valenciennes, 1831)	O	Fricke 1999* Remarks: Records from Mauritius possibly subtidal; intertidal record from Réunion.	
*Parupeneus barberinus* (Lacepède, 1801)	O	Ntiba et al. 1993* Remark: Records in the lagoon of Mauritius (Fricke 1999) are below the low tide line.	
*Parupeneus ciliatus* (Lacepède, 1802)	O	Fricke 1999* Remarks: No detailed locality published from Mauritius; intertidal record from Réunion.	
*Parupeneus heptacanthus* (Lacepède, 1802)	O	Ntiba et al. 1993* (as *P. cinabarinus*), Fricke 1999 (as *P. bifasciatus*) Remark: Only subtidal records published from Mauritius (Fricke 1999).	
*Parupeneus indicus* (Russell in Shaw, 1803)	O	Ntiba et al. 1993* Remark: No detailed locality published from Mauritius (Fricke 1999).	
*Parupeneus macronemus* (Lacepède, 1801)	O	Ntiba et al. 1993* Remark: Records from Mauritius possibly subtidal (Fricke 1999).	
*Parupeneus trifasciatus* (Lacepède, 1801)	O	Fricke 1999* (as *P. bifasciatus*) Remarks: Records from Mauritius possibly subtidal; intertidal record from Réunion.	
*Upeneus vittatus* (Forsskål in Niebuhr, 1775)	O	Ntiba et al. 1993* Remark: No detailed locality published from Mauritius (Fricke 1999).	
**Muraenidae Rafinesque, 1815 – Moray eels**			
*Anarchias seychellensis* Smith, 1962	R	Fricke 1999 Remarks: Often found in tidepools (Froese and Pauly 2018). Records in Mauritius subtidal.	
*Echidna nebulosa* (Ahl, 1789)	**R**!	Fricke 1999, Fricke et al. 2013*, Sindorf et al. 2015*, EA 2017, 2018	USNM 342098 (1), 342099 (1)
*Echidna polyzona* (Richardson, 1845)	R	Fricke 1999	USNM 342101 (2)
*Gymnothorax buroensis* (Bleeker, 1857)	R	Fricke 1999 Remark: Primarily found in the surge zone (Lieske and Myers 1994).	USNM 3442112 (4)
*Gymnothorax chilospilus* Bleeker, 1864	R	Fricke 1999, Fricke et al. 2009*	USNM 342118 (1)
*Gymnothorax enigmaticus* McCosker & Randall, 1982	O	Fricke 1999 Remark: May occur in intertidal reefs (Kuiter and Tonozuka 2001).	
*Gymnothorax eurostus* (Abbott, 1860)	O	Fricke 1999, Fricke et al. 2009*	USNM 342121 (1)
*Gymnothorax fimbriatus* (Bennett, 1832)	O	Fricke et al. 2009* Remarks: Young specimens in tidepools (Fricke et al. 2009). Mauritian records subtidal (Fricke 1999).	
*Gymnothorax flavimarginatus* (Rüppell, 1830)	O	Fricke 1999, Fricke et al. 2009* Remark: Young specimens in tidepools (Fricke et al. 2009).	USNM 342130 (4), 342131 (3), 342132 (1)
*Gymnothorax griseus* (Lacepède, 1803)	O	Fricke 1999, Fricke et al. 2013*, EA 2018	USNM 342258 (1), 342260 (1)
*Gymnothorax javanicus* (Bleeker, 1859)	O	Fricke et al. 2009* Remark: No detailed locality published from Mauritius (Fricke 1999).	
*Gymnothorax johnsoni* (Smith, 1962)	O	Fricke et al. 2009* Remark: Only subtidal records known from Mauritius (Fricke 1999).	
*Gymnothorax margaritophorus* Bleeker, 1864	O	Fricke 1999	USNM 342142 (1)
*Gymnothorax melatremus* Schultz, 1953	O	Fricke 1999	USNM 342147 (1)
*Gymnothorax meleagris* (Shaw, 1795)	O	Fricke 1999, Fricke et al. 2009* Remarks: No detailed locality published from Mauritius. Young specimens in tidepools (Fricke et al. 2009).	SMNS 16899 (1)
*Gymnothorax pictus* (Ahl, 1789)	R	Durville and Chabanet 2009*, Fricke et al. 2009*, Fricke et al. 2013* Remarks: Juvenile and adult specimens in tidepools (Durville and Chabanet 2009); R acc. to Durville and Chabanet (2009) and Sindorf et al. (2015). Only one record without depth published from Mauritius (Fricke 1999).	
*Gymnothorax phasmatodes* (Smith, 1962)	O	Allen and Erdmann 2012* Remark: Only subtidal records known from Mauritius (Fricke 1999).	
*Gymnothorax rueppelliae* (McClelland, 1844)	O	Fricke 1999, Fricke et al. 2009*, Fricke et al. 2013* Remarks: Young specimens in shallow water and tidal pools (Fricke et al. 2009).	SMNS 16921 (1), USNM 342263 (1), 342264 (1)
*Gymnothorax undulatus* (Lacepède, 1803)	O	Fricke et al. 2009* Remarks: Young specimens in shallow water and tidal pools (Fricke et al. 2009). Mauritian records subtidal (Fricke 1999).	
*Strophidon sathete* (Hamilton, 1822)	R	Randall et al. 1990* Remarks: In estuarine areas (Randall et al. 1990). No detailed locality published from Mauritius (Fricke 1999).	
*Uropterygius macrocephalus* (Bleeker, 1864)	O	Fricke 1999	USNM 342094 (8)
**Ophichthidae Günther, 1870 – Snake eels**			
*Leiuranus semicinctus* (Lay & Bennett, 1839)	O	Fricke 1999	USNM 342249 (1)
*Muraenichthys schultzei* Bleeker, 1857	O	Fricke 1999 Remark: Mauritian records in 0-1 m (in tidepools and in mouth of a stream with mangrove shore).	USNM 342245 (1), 342246 (1)
*Myrichthys maculosus* (Cuvier, 1816)	O	Fricke 1999, Fricke et al. 2013* Remarks: Mauritian records subtidal, 1.5 m or deeper (Fricke 1999) or in the lagoon below low tide line (EA 2018).	
*Scolecenchelys robusta* Hibino & Kimura, 2015	?O/R!	Fricke 1999 (as *Muraenichthys laticaudatus*) Remarks: Mauritian records in tidepools and deeper to 8 m. Taxonomy follows Hibino and Kimura (2016).	SMNS 16890 (1 paratype), USNM 342241 (1), 342244 (2)
*Yirrkala tenuis* (Günther, 1870)	T	Keith et al. 1999* Remark: No detailed locality published from Mauritius (Fricke 1999).	
**Ophidiidae Rafinesque, 1810 - Cusk eels**			
*Brotula multibarbata* Temminck & Schlegel, 1846	T	Roux 2013* Remark: No detailed locality published from Mauritius (Fricke 1999).	
**Ostraciidae Rafinesque, 1810**			
*Lactoria cornuta* (Linnaeus, 1758)	T	Ntiba et al. 1993*, Fricke et al. 2009* Remarks: In mangrove creeks, in estuaries and harbours (Ntiba et al. 1993, Kuiter and Tonozuka 2001). Records from Mauritius possibly subtidal (Fricke 1999).	
*Ostracion cubicus* Linnaeus, 1758	T	Fricke 1999	USNM 348109 (1)
**Plesiopidae Günther, 1861 – Longfins, roundheads**			
*Plesiops coeruleolineatus* Rüppell, 1835	**R**!	Fricke 1999 Remarks: Under rubble and stones in flood basins and pools (Kuiter and Tonozuka 2001, Masuda and Allen 1993). Mauritian tidepool records from Albion and Pointe aux Caves (leg. Heemstra et al. 1995, USNM material).	USNM 343772 (3), 343774 (8)
*Plesiops mystaxus* Mooi, 1995	?**R**!/O	Fricke 1999 Remark: Tidepool records from Pointe aux Caves (leg. Heemstra et al. 1995, USNM material).	USNM 343787 (1)
**Plotosidae Bleeker, 1858 – Eel catfishes**			
*Plotosus lineatus* (Thunberg, 1787)	O	Fricke 1999, Fricke et al. 2009*, Sindorf et al. 2015*	USNM 350022 (2)
**Polynemidae Rafinesque, 1815 – Threadfins**			
*Leptomelanosoma indicum* (Shaw, 1804)	T	Fricke et al. 2009* Remarks: Inshore, including tidepools, estuaries and lower reaches of streams (Fricke et al. 2009). No detailed locality published from Mauritius so far.	
*Polydactylus plebeius* (Broussonet, 1782)	T	Fricke 1999 Remark: USNM material from a mangrove area.	USNM 349503 (1)
**Pomacanthidae Jordan & Evermann, 1898 – Angelfishes**			
*Pomacanthus semicirculatus* (Cuvier, 1831)	O	Fricke et al. 2009* Remarks: T acc. to Murase (2015). No detailed locality published from Mauritius (Fricke 1999).	
**Pomacentridae Bonaparte, 1831** – **Damselfishes**			
*Abudefduf margariteus* (Cuvier, 1830)	O	Fricke et al. 2009* Remark: Records from Mauritius possibly subtidal (Fricke 1999).	
*Abudefduf septemfasciatus* (Cuvier, 1830)	O	Fricke 1999, Durville and Chabanet 2009*, EA 2017, 2018	USNM 346062 (7), 342063 (30), 346064 (5)
*Abudefduf sexfasciatus* (Lacepède, 1801)	O	Sindorf et al. 2015* Remarks: R acc. to Sindorf et al. (2015); all species of *Abudefduf* are O acc. to Cox et al. (2011); SMNS material, Taiwan. So far, only subtidal records known from Mauritius (Fricke 1999).	
*Abudefduf sordidus* (Forsskål in Niebuhr, 1775)	O	Fricke 1999, Lieske and Myers 2004*, Durville and Chabanet 2009*, Fricke et al. 2009*, Fricke et al. 2013*, Roux 2013*, EA 2017, 2018 Remark: O acc. to Durville and Chabanet (2009); T acc. to Murase (2015).	SMNS 16881 (2), 16905 (2), USNM 346055 (4), 346067 (6), 346068 (13)
*Abudefduf sparoides* (Quoy & Gaimard, 1825)	O	Fricke 1999, Fricke et al. 2009*, Fricke et al. 2013* Remark: R acc. to Sindorf et al. (2015).	USNM 346056 (8), 346060 (2)
*Abudefduf vaigiensis* (Quoy & Gaimard, 1825)	O	Durville and Chabanet 2009*, Fricke et al. 2013*, Roux 2013* Remarks: O acc. to Lieske and Myers (2004) and Durville and Chabanet (2009); R acc. to Sindorf et al. (2015); T acc. to Griffiths (2003b) and Murase (2015). No detailed locality published from Mauritius (Fricke 1999).	
*Chromis viridis* (Cuvier, 1830)	O	Fricke et al. 2009* Remark: Known records from Mauritius are subtidal (Fricke 1999).	
*Chrysiptera biocellata* (Quoy & Gaimard, 1825)	O	Fricke 1999, Durville and Chabanet 2009*, Sindorf et al. 2015*	USNM 346016 (1)
*Chrysiptera brownriggii* (Bennett, 1828)	O	Fricke 1999 (as *C. leucopoma*), Fricke et al. 2013*, EA 2017, 2018 Remarks: R acc. to Sindorf et al. 2015, T acc. to Murase (2015).	
*Chrysiptera glauca* (Cuvier, 1830)	O	Fricke 1999, Durville and Chabanet 2009*, Fricke et al. 2009*, Fricke et al. 2013*, EA 2017, 2018 Remarks: O acc. to Durville and Chabanet (2009); R acc. to Sindorf et al. (2015); T acc. to Murase (2015).	USNM 346019 (1), 346021 (4)
*Chrysiptera unimaculata* (Cuvier, 1830)	O	Fricke 1999, Fricke et al. 2013*	USNM 346026 (1)
*Dascyllus carneus* Fischer, 1885	O	Ntiba et al. 1993* Remarks: Kenyan intertidal record in a mangrove creek; no detailed locality published from Mauritius (Fricke 1999).	
*Dascyllus trimaculatus* (Rüppell, 1829)	O	Ntiba et al. 1993* Remarks: Kenyan intertidal record in a mangrove creek; only subtidal records published from Mauritius (Fricke 1999).	
*Plectroglyphidodon imparipennis* (Vaillant & Sauvage, 1875)	O	Fricke 1999, Fricke et al. 2013* Remark: R acc. to Cox et al. (2011).	USNM 345725 (2)
*Plectroglyphidodon johnstonianus* Fowler & Ball, 1924	O	Fricke 1999, Fricke et al. 2009* Remarks: Known records from Mauritius are subtidal; intertidal record from Réunion.	
*Plectroglyphidodon leucozonus* (Bleeker, 1859)	O	Fricke 1999 Remark: T acc to Murase (2015).	USNM 346040 (9), 346042 (4)
*Plectroglyphidodon phoenixensis* (Schultz, 1943)	O	Fricke 1999, Fricke et al. 2013*	USNM 346043 (1)
*Plectroglyphidodon randalli* Allen, 1991	O	Fricke 1999 (listed as *P. leucozonus*).	USNM 346044 (10)
*Pomacentrus agassizii* Bliss, 1883	O	Fricke 1999	SMNS 16895 (1), 16915 (1)
*Pomacentrus caeruleus* Quoy & Gaimard, 1825	O	Sindorf et al. 2015* Remark: Known records in Mauritius are below low tide line or much deeper (Fricke 1999).	
*Stegastes luteobrunneus* (Smith, 1960)	O	Fricke 1999 (as *S. fasciolatus*), EA 2018 Remark: *Stegastes* sp = O acc. to González-Murcia et al. (2016).	USNM 347015 (1)
*Stegastes limbatus* (Cuvier, 1830)	**R**!	Fricke 1999, EA 2018 Remark: Several tidepool records from Mauritius (leg. Heemstra et al. 1995, USNM material).	USNM 347001 (6), 347002 (7), 347003 (16)
*Stegastes nigricans* (Lacepède, 1802)	O	Fricke 1999* Remarks: Intertidal record from Réunion. Records from Mauritius possibly subtidal.	
*Stegastes pelicieri* Allen & Emery, 1985	O	Fricke 1999* Remarks: Intertidal record from Réunion. Records from Mauritius possibly subtidal.	
*Stegastes punctatus* (Quoy & Gaimard, 1825)	O	Fricke 1999* Remarks: Intertidal record from Réunion. No detailed locality published published from Mauritius.	
**Pseudochromidae Müller & Troschel, 1849 – Dottybacks, eel blennies**			
*Haliophis guttatus* (Forsskål in Niebuhr, 1775)	R	Durville and Chabanet 2009* Remark: No detailed locality published from Mauritius (Fricke 1999).	
**Scaridae Rafinesque, 1810 – Parrotfishes**			
*Leptoscarus vaigiensis* (Quoy & Gaimard, 1824)	O	Ntiba et al. 1993* Remark: Records from Mauritius possibly subtidal (Fricke 1999).	
*Scarus ghobban* Forsskål in Niebuhr, 1775	O	Fricke 1999* Remarks: No intertidal record known from Mauritius; intertidal record from Réunion.	
*Scarus psittacus* Forsskål in Niebuhr, 1775	O	Ntiba et al. 1993* Remark: Kenyan intertidal records in mangrove creeks; no detailed locality published from Mauritius (Fricke 1999).	
**Scorpaenidae Risso, 1827 – Scorpionfishes**			
*Caracanthus madagascariensis* (Guichenot, 1869)	?O/T	Fricke 1999	USNM 349960 (1)
*Dendrochirus zebra* (Cuvier, 1829)	T	Fricke 1999	SMNS 16938 (1), USNM 349895 (2), 349897 (1)
*Pterois miles* (Bennett, 1828)	T	Ntiba et al. 1993*, Fricke 1999	USNM 349904 (1)
*Scorpaenodes guamensis* (Quoy & Gaimard, 1824)	?O/T	Fricke et al. 2009*, Fricke et al. 2013* Remark: No detailed locality published from Mauritius (Fricke 1999).	
*Scorpaenodes parvipinnis* (Garrett, 1864)	?O/T	Fricke et al. 2009*, Fricke et al. 2013* Remark: No detailed locality published from Mauritius (Fricke 1999).	SMNS 16927 (1)
*Scorpaenopsis gibbosa* (Bloch & Schneider, 1801)	?O/T	Fricke et al. 2009* Remark: No confirmed intertidal record from Mauritius so far (Fricke 1999).	
*Sebastapistes mauritiana* (Cuvier, 1829)	R	Fricke 1999, Fricke et al. 2009* Remark: In outer intertidal reef flats, lagoons and pools exposed to wave action.	SMNS 16914 (1), USNM 349972 (3)
*Sebastapistes strongia* (Cuvier, 1829)	?R/O	Fricke 1999	SMNS 16910 (1)
*Sebastapistes tinkhami* (Fowler, 1946)	?O/T	Fricke 1999	USNM 349975 (6)
**Serranidae Swainson, 1839 – Groupers**			
*Cephalopholis argus* Schneider, 1801	O	Fricke 1999, Fricke et al. 2009*, Fricke et al. 2013* Remarks: T acc. to Murase (2015). Juveniles are found in tidepools (Heemstra and Randall 1993).	USNM 349591 (1)
*Cephalopholis boenak* (Bloch, 1790)	O	SMNS material* (Taiwan) Remark: No detailed locality published from Mauritius (Fricke 1999).	
*Cephalopholis urodeta* (Forster, 1801)	O	Fricke 1999	USNM 349605 (1)
*Epinephelus coeruleopunctatus* (Bloch, 1790)	O	Fricke et al. 2009*, SMNS material* (Australia) Remarks: No detailed locality published from Mauritius (Fricke 1999). Juveniles are found in tidepools (Heemstra and Randall 1993).	
*Epinephelus hexagonatus* (Forster in Bloch & Schneider, 1801)	O	Fricke 1999, Fricke et al. 2009*, Fricke et al. 2013*	SMNS 16926 (1), USNM 349558 (8), 349559 (1), 349562 (2)
*Epinephelus merra* Bloch, 1793	O	Fricke 1999, Fricke et al. 2009*, Fricke et al. 2013*	USNM 349565 (11)
*Epinephelus rivulatus* (Valenciennes, 1830)	O	Roux 2013* Remark: No detailed locality published from Mauritius (Fricke 1999).	
*Epinephelus spilotoceps* Schultz, 1953	O	Fricke et al. 2009*, SMNS material* (Australia) Remark: No detailed locality published from Mauritius (Fricke 1999).	
*Epinephelus tauvina* (Forsskål in Niebuhr, 1775)	O	Fricke 1999, Fricke et al. 2009* Remark: Juveniles may occur in tidepools (Heemstra and Randall 1993).	SMNS 16901 (1), USNM 349572 (12), 349573 (1), 349574 (5)
*Epinephelus tukula* Morgans, 1959	O	Fricke et al. 2009* Remarks: Juveniles may occur in tidepools (Heemstra and Randall 1993). No detailed locality from Mauritius published so far (Fricke 1999).	
*Grammistes sexlineatus* (Thunberg, 1792)	O	Fricke 1999, Fricke et al. 2013*, Sindorf et al. 2015* Remark: T acc. to Murase (2015).	SMNS 16906 (1), USNM 349546 (6), 349550 (1), 349551 (3)
*Pseudogramma polyacantha* (Bleeker, 1856)	O	Fricke et al. 2009* Remark: So far, only tidepool records from Rodrigues are known.	
**Siganidae Richardson, 1837** – **Rabbitfishes**			
*Siganus sutor* (Valenciennes, 1835)	?O/T	Ntiba et al. 1993*, Fricke 1999	SMNS 16897 (1)
*Siganus laqueus* Bonde, 1934	O	Woodland 1986*, Fricke et al. 2009* Remarks: Juveniles enter weedy estuaries (Woodland 1986). No detailed locality from Mauritius published so far (Fricke 1999).	
**Soleidae Bonaparte, 1833 – Soles**			
*Pardachirus marmoratus* (Lacepède, 1802)	T	Ntiba et al. 1993* Remarks: In intertidal mangrove creeks; Mauritian records in shallow lagoon, but below the low tide line (Fricke 1999, EA 2017).	
**Synanceiidae Swainson, 1839** – **Stonefishes**			
*Synanceia verrucosa* Bloch & Schneider, 1801	?O/T	Fricke et al. 2009* Remark: No confirmed intertidal record from Mauritius so far (Fricke 1999).	
**Syngnathidae Bonaparte, 1831 – Seahorses, pipefishes**			
*Choeroichthys valencienni* Kaup, 1856	?O/T	Fricke et al. 2009* Remark: Mauritian records subtidal or deeper (Fricke 1999).	
*Corythoichthys flavofasciatus* (Rüppell, 1838)	?O/T	Fricke 1999, Fricke et al. 2009* Remarks: Records from Mauritius possibly subtidal; intertidal record from Réunion.	
*Doryrhamphus bicarinatus* Dawson, 1981	?O/T	Fricke 1999 (as *D. excisus*), Fricke et al. 2009* Remark: Records from Mauritius possibly subtidal.	
*Halicampus mataafae* (Jodan & Seale, 1906)	?O/T	Fricke et al. 2009* Remark: Known records from Mauritius are subtidal (Fricke 1999).	
*Hippichthys cyanospilus* (Bleeker, 1854)	R	Dawson 1986*, Fricke 1999, Kuiter and Tonozuka 2001* Remarks: In estuaries, mangroves and freshwater of coastal streams; Mauritian records from a mangrove area (leg. Heemstra et al. 1995, USNM material).	USNM 348075 (1)
*Hippichthys spicifer* (Rüppell, 1838)	R	Fricke 1999, Allen et al. 2002* Remarks: In tidal creeks, estuaries, mangroves and freshwater of coastal streams; Mauritian records from a mangrove area (leg. Heemstra et al. 1995, USNM material).	USNM 348076 (2)
*Microphis millepunctatus* (Kaup, 1856)	R	Fricke 1999, Dawson 1986* Remarks: In estuaries, mangroves and freshwater of coastal streams; Mauritian records from a mangrove area (leg. Heemstra et al. 1995, USNM material).	USNM 348078 (4)
*Nannocampus pictus* (Duncker, 1915)	?O/T	Fricke 1999, Fricke et al. 2009*	SMNS 16902 (2)
*Phoxocampus belcheri* (Kaup, 1856)	T	Fricke 1999 Remark: T acc. to Murase (2015).	USNM 348072 (1)
**Synodontidae Gill, 1861 – Lizardfishes**			
*Saurida gracilis* (Quoy & Gamard, 1824)	?O/T	Ntiba et al. 1993*, Fricke 1999 Remark: Intertidal records from mangrove areas.	USNM 349534 (2)
*Synodus variegatus* (Lacepède, 1803)	?T/O	Fricke 1999 Remarks: Intertidal record from Réunion; found on reef flat, 0-1 m in Mauritius.	
*Trachinocephalus trachinus* (Temminck & Schlegel, 1846)	?T/O	Ntiba et al. 1993* (as *T. myops*), Fricke et al. 2009* (as *T. myops*) Remarks: In estuaries and intertidal mangrove creeks. No detailed locality published from Mauritius (Fricke 1999).	
**Terapontidae Richardson, 1842 – Thornfishes**			
*Terapon jarbua* (Forsskål in Niebuhr, 1775)	O	Ntiba et al. 1993*, Fricke et al. 2009* Remarks: Kenyan record in an intertidal mangrove creek; no detailed locality published from Mauritius (Fricke 1999).	
**Tetraodontidae Bonaparte, 1831** – **Pufferfishes**			
*Amblyrhynchotes honckenii* (Bloch, 1775)	O	Beckley 1985*, Smith and Heemstra 1986* Remarks: Common in tidepools and estuaries (Smith and Heemstra 1986). Record in Mauritius questionable (Fricke 1999).	
*Arothron immaculatus* (Bloch & Schneider, 1801)	O	Ntiba et al. 1993*, Fricke 1999 Remark: Intertidal records from mangrove areas.	USNM 348088 (7)
*Canthigaster amboinensis* (Bleeker, 1864)	O	Fricke 1999	SMNS 16896 (1), USNM 48090 (1), 348092 (1)
*Canthigaster janthinoptera* (Bleeker, 1855)	O	Fricke 1999, Fricke et al. 2009* Remarks: Records from Mauritius possibly subtidal; intertidal record from Réunion.	
*Canthigaster natalensis* (Günther, 1870)	O	Fricke 1999	USNM 348095 (2), 348096 (1), 348098 (8)
*Canthigaster solandri* (Richardson, 1845)	O	Fricke et al. 2009*; Sindorf et al. 2015* Remarks: May occur in intertidal areas (Fricke et al. 2009), such as reef flats (Froese and Pauly 2018) and intertidal seagrass meadows (Arndt, unpubl. obs.). No detailed locality published from Mauritius (Fricke 1999).	
*Canthigaster valentini* (Bleeker, 1853)	O	Fricke 1999, Fricke et al. 2009* Remark: Records from Mauritius possibly subtidal.	
**Tripterygiidae Whitley, 1931 – Triplefin blennies**			
*Enneapterygius abeli* (Klausewitz, 1960)	**R**!	Fricke 1999, Fricke et al. 2013* Remark: Mauritian records from the surge zone and tidepools (leg. Heemstra et al. 1995, USNM material).	USNM 344032 (8), 344070 (1)
*Enneapterygius elegans* (Peters, 1876)	?R/O	Fricke 1999, Fricke et al. 2009*	
*Enneapterygius philippinus* (Peters, 1868)	**R**!	Fricke 1999, Fricke et al. 2009*, Fricke et al. 2013* Remarks: T acc. to Murase (2015). Several tidepool records from Mauritius (leg. Heemstra et al. 1995, USNM material).	SMNS 16892 (2), 16911 (19), 16937 (16), USNM 343957 (1), 343959 (1), 343960 (1)
*Enneapterygius tutuilae* Jordan & Seale, 1906	?R/O	Fricke 1999, Fricke et al. 2009*	
*Helcogramma alkamr* Holleman, 2007	R	Holleman 2007*, Fricke et al. 2009* (as *H. obtusirostris*) Remarks: In high energy tidal environments (Holleman 2007). Records from Mauritius possibly subtidal (Fricke 1999).	
*Helcogramma fuscopinna* Holleman, 1982	R	Fricke 1999, Fricke et al. 2009* Remark: In Mauritius on rocks of the surge zone.	

**Table 2.** Permanent resident species in 23 examined shallow Mauritian tidepools in the year 2018. Location of records: AL – Albion; BB – Blue Bay; LH – Lighthouse at Pointe aux Caves; PE – Péreybère.

**Species**	**N (pools)**	**Locations**	**Remarks**
*Alticus monochrus*	7	AL	Always single individuals in the pool, whereas lots of individuals were active on the seaward rocks.
*Bathygobius coalitus*	20	AL, BB, LH, PE	By far the most abundant species in BB, reaching abundances up to 5 ind./m^2^. Also occurring in the smallest and shallowest pools.
*Blenniella cf. periophthalmus*	2	AL	Few individuals. Also recorded in BB in the year 2017.
*Echidna nebulosa*	2	AL	Single individuals. Also recorded in BB in the year 2017.
*Istiblennius bellus*	11	AL, LH	Very abundant species in AL and LH; reaching abundances up to 2.5 ind./m^2^. Also recorded in BB in the year 2017.
*Istiblennius edentulus*	22	AL, BB, LH, PE	Very abundant species in BB, AL and PE; reaching abundances up to 4 ind./m^2^ in BB and 2.5 ind./m^2^ in AL.
*Istiblennius dussumieri*	1	BB	Single individual.
*Stegastes limbatus*	3	AL, BB	One or two individuals near large boulders, defending their territory aggressively.

## References

[B5239174] Able Kenneth W. (2005). A re-examination of fish estuarine dependence: Evidence for connectivity between estuarine and ocean habitats. Estuarine, Coastal and Shelf Science.

[B5239184] Aguilar-Perera A, Appeldoorn RS (2007). Variation in juvenile fish density along the mangrove seagrass coral reef continuum in SW Puerto Rico. Marine Ecology Progress Series.

[B5239194] Allen G. R. (1991). Field Guide to the Freshwater Fishes of New Guinea.

[B5239203] Allen G. R., Midgley H., Allen M. (2002). Field Guide to the Freshwater Fishes of Australia.

[B5243326] Allen G. R., Erdmann M. V. (2012). Reef Fishes of the East Indies.

[B5239222] Beckley Lynnath E. (1985). The fish community of East Cape tidal pools and an assessment of the nursery function of this habitat. South African Journal of Zoology.

[B5239242] Bennett B., Griffiths C. L., Penrith Mary-Louise (1983). The diets of littoral fish from the Cape Peninsula. South African Journal of Zoology.

[B5239232] Bennett B. A., Griffiths C. L. (1984). Factors affecting the distribution, abundance and diversity of rock-pool fishes on the Cape Peninsula, South Africa. South African Journal of Zoology.

[B5239252] Berry PF, Elst RP, vander Hanekom P, Joubert CSW, Smale MJ (1982). Density and biomass of the ichthyofauna of a Natal littoral reef. Marine Ecology Progress Series.

[B5239273] Bhikajee M (1996). Growth, reproductive biology and behaviour of the amphibious blenny *Alticus
monochrus* (Pisces, Blenniidae) on the island of Mauritius.

[B5239263] Bhikajee M., Green J. M. (2002). Behaviour and habitat of the Indian Ocean amphibious blenny, *Alticus
monochrus*. African Zoology.

[B5239282] Bhikajee M., Green J. M., Dunbrack R. (2006). Life history characteristics of *Alticus
monochrus*, a supratidal blenny of the southern Indian Ocean. African Zoology.

[B5239292] Bianchi G. (1985). FAO species identification sheets for fishery purposes. Field guide to the commercial marine and brackish-water species of Pakistan. Prepared with the support of PAK / 77 / 033 / and FAO (FIRM) Regular Programme.

[B5297420] Burger L. F. (1990). The Distribution Patterns and Community Structure of the Tsitsikamma Rocky Littoral Ichthyofauna..

[B5239301] Chotkowski Michael A., Buth Donald G., Prochazka Kim (1999). Systematics of intertidal fishes. In: Horn MH, Martin KLM, Chotkowski MA (Eds) Intertidal fishes. Life in two worlds. Academic Press, San Diego,.

[B5239351] Cox Traci Erin, Baumgartner Erin, Philippoff Joanna, Boyle Kelly S. (2011). Spatial and vertical patterns in the tidepool fish assemblage on the island of O'ahu. Environmental Biology of Fishes.

[B5239371] Dawson CE, Daget J, Gosse J-P, Thys van den Audenaerde DFE (1986). Syngnathidae. Check-list of the freshwater fishes of Africa (CLOFFA).

[B5239385] Durville P, Chabanet P (2009). Intertidal rock pool fishes in the Natural Reserve of Glorieuses Islands (Western Indian Ocean). Western Indian Ocean Journal of Marine Science.

[B5239395] Duvat V. (2009). Beach erosion management in small island developing states: Indian Ocean case studies. In: Brebbia CA, Benassai G, Rodriguez G (Eds) Coastal Processes. WIT Transactions on Ecology and the Environment.

[B5239405] Fagoonee I. (1990). Coastal marine ecosystems of Mauritius. Hydrobiologia.

[B5239415] Figueiredo G. G. A. A., Pessanha A. L. M. (2016). Comparative study of trophic organization of juvenile fish assemblages of three tidal creeks in a tropical semi-arid estuary. Journal of Fish Biology.

[B5239426] Fricke Ronald A. (1986). Family No. 239: Callionymidae. In: Smith MM, Heemstra PP (Eds) Smith’s sea fishes.

[B5239440] Fricke R. (1999). Fishes of the Mascarene Islands (Réunion, Mauritius, Rodriguez). An annotated checklist, with descriptions of new species.

[B5239468] Fricke R, Mulochau T, Durville P, Chabanet P, Tessier E, Letourneur Y (2009). Annotated checklist of the fish species (Pisces) of La Réunion, including a Red List of threatened and declining species. Stuttgarter Beiträge zur Naturkunde A,.

[B5239449] Fricke R, Durville P, Bernardi G, Borsa P, Mou-Tham G, Chabanet P (2013). Checklist of the shore fishes of Europa Island, Mozambique Channel, southwestern Indian Ocean, including 302 new records. Stuttgarter Beiträge zur Naturkunde A,.

[B5239459] Fricke R, Eschmeyer WN, van der Laan R (Eds) Eschmeyer's Catalog of Fishes: Genera, Species, References. http://researcharchive.calacademy.org/research/ichthyology/catalog/fishcatmain.asp.

[B5239480] Froese R, Pauly D FishBase. World Wide Web electronic publication, version 12/2018. http://www.fishbase.se/search.php.

[B5239489] Ghanbarifardi Mehdi, Malek Masoumeh (2009). Distribution, diversity, and abundance of rocky intertidal fishes in the Persian Gulf and Gulf of Oman, Iran. Marine Biology Research.

[B5239311] Gibson R. N. (1999). Methods for Studying Intertidal Fishes. In: Horn MH, Martin KLM, Chotkowski MA (Eds) Intertidal fishes. Life in two worlds. Academic Press, San Diego,.

[B5239321] Gibson R. N., Yoshiyama R. M. (1999). Intertidal Fish Communities. In: Horn MH, Martin KLM, Chotkowski MA (Eds) Intertidal fishes. Life in two worlds. Academic Press, San Diego,.

[B5239499] Gill AC (1998). *Hetereleotris
georgegilli*, a new species of gobiid fish, with comments on other Mauritian *Hetereleotris*. Bulletin of the Natural History Museum, London (Zoology).

[B5239509] González-Murcia S, Chicas Batres F, Lovo MH (2016). Community structure and height distribution of intertidal rockpool fish in Los Cobanos, El Salvador. Pan-American Journal of Aquatic Sciences.

[B5297355] Griffiths S. P. (2003). Rockpool ichthyofaunas of temperate Australia: species composition, residency and biogeographic patterns. Estuarine, Coastal and Shelf Sciences.

[B5298309] Griffiths S. P. (2003). Spatial and temporal dynamics of temperate Australian rockpool ichthyofaunas. Marine and Freshwater Research.

[B5297375] Hammer Ø., Harper D. A.T., Ryan P. D. (2001). PAST: Paleontological Statistics Software Package for Education and Data Analysis. Palaeontologia Electronica.

[B5239519] Harrison IJ, Senou H, Carpenter KE, Niem VH (1999). Mugilidae. FAO species identification guide for fishery purposes. The living marine resources of the Western Central Pacific.

[B5239533] Heemstra PC, Randall JE (1993). FAO Species Catalogue. Vol. 16. Groupers of the world (family Serranidae, subfamily Epinephelinae). An annotated and illustrated catalogue of the grouper, rockcod, hind, coral grouper and lyretail species known to date.

[B5239542] Hibino Yusuke, Kimura Seishi (2016). Revision of the *Scolecenchelys
gymnota* species group with descriptions of two new species (Anguilliformes: Ophichthidae: Myrophinae). Ichthyological Research.

[B5239552] Holleman W. (2007). Fishes of the genus *Helcogramma* (Blennioidei: Tripterygiidae) in the Western Indian Ocean, including Sri Lanka, with descriptions of four new species. Smithiana Bulletin.

[B5239154] Horn MH, Martin KLM, Chotkowski MA (1999). Intertidal fishes: life in two worlds. Academic Press, San Diego,.

[B5239331] Horn M. H., Ojeda F. P. (1999). Herbivory. In: Horn MH, Martin KLM, Chotkowski MA (Eds) Intertidal fishes. Life in two worlds. Academic Press, San Diego,.

[B5239562] Ikejima K, Tongnunui P, Medej T, Taniuchi T (2003). Juvenile and small fishes in a mangrove estuary in Trang province, Thailand: seasonal and habitat differences. Estuarine, Coastal and Shelf Science.

[B5239572] Jaxion-Harm Jessica, Saunders James, Speight Martin R. (2012). Distribution of fish in seagrass, mangroves and coral reefs: life-stage dependent habitat use in Honduras. Revista de Biología Tropical.

[B5239582] Jones GP, Andrew NL (1990). Herbivory and patch dynamics on rocky reefs in temperate Australasia: The roles of fish and sea urchins. Australian Journal of Ecology.

[B5239592] Keith P, Vigneux E, Bosc P (1999). Atlas des poissons et des crustacés d’eau douce de La Réunion..

[B5297385] Krebs C. J. (1999). Ecological Methodology.

[B5239601] Kuiter RH, Tonozuka T (2001). Pictorial guide to Indonesian reef fishes. Part 1. Eels- Snappers, Muraenidae - Lutjanidae..

[B5242536] Laegdsgaard Pia, Johnson Craig (2001). Why do juvenile fish utilise mangrove habitats?. Journal of Experimental Marine Biology and Ecology.

[B5239620] Lieske E, Myers R (1994). Coral reef fishes. Indo-Pacific & Caribbean including the Red Sea.

[B5239629] Lieske E, Myers R (2004). Coral reef guide, Red Sea.

[B5239638] Lundquist CJ, Pinkerton MH (2008). Collation of data for ecosystem modelling of Te Tapuwae o Rongokako Marine Reserve.

[B5239647] Mahon Robin, Mahon Susan D. (1994). Structure and resilience of a tidepool fish assemblage at Barbados. Environmental Biology of Fishes.

[B5239341] Martin Karen L. M., Bridges Christopher R. (1999). Respiration in water and air. In: Horn MH, Martin KLM, Chotkowski MA (Eds) Intertidal fishes. Life in two worlds. Academic Press, San Diego,.

[B5239676] Masuda Hajime, Amaoka Kunio, Araga Chuichi, Uyeno T., Yoshino Tetsuo (1984). The fishes of the Japanese Archipelago. Vol. 1. Tokai University Press, Tokyo, Japan.

[B5239667] Masuda H, Allen GR (1993). Meeresfische der Welt - Groß-Indopazifische Region.

[B5239701] Maugé LA, Daget J, Gosse J, Thys van den Audenaerde D (1986). Gobiidae. Check-list of the freshwater fishes of Africa (CLOFFA).

[B5239657] McClanahan TR, Maina J, Moothien-Pillay R, Baker AC (2005). Effects of geography, taxa, water flow, and temperature variation on coral bleaching intensity in Mauritius. Marine Ecology Progress Series.

[B5239715] Miller J. M., Crowder L. B., Moser M. L. (1985). Migration and utilization of estuarine nurseries by juvenile fishes, an evolutionary perspective. Contributions in Marine Science.

[B5239725] Montaggioni L. F., Faure G. (1997). Response of reef coral communities to sea-level rise: a Holocene model from Mauritius (Western Indian Ocean). Sedimentology.

[B5239735] Mumby P. J., Edwards A. J., Arias-González J. E., Lindeman K. C., Blackwell P. G., Gall A., Gorczynska M. I., Harborne A. R., Pescod C. L., Renken H., Wabnitz C. C.C., Llewellyn G. (2004). Mangroves enhance the biomass of coral reef fish communities in the Caribbean. Nature.

[B5239829] Murase Atsunobu (2013). Community structure and short temporal stability of a rockpool fish assemblage at Yaku-shima Island, southern Japan, northwestern Pacific. Ichthyological Research.

[B5243335] Murase Atsunobu (2015). Ichthyofaunal diversity and vertical distribution patterns in the rockpools of the southwestern coast of Yaku-shima Island, southern Japan. Check List.

[B5239753] Nagelkerken I., van der Velde G., Gorissen M. W., Meijer G. J., Van't Hof T., den Hartog C. (2000). Importance of mangroves, seagrass beds and the shallow coral reef as a nursery for important coral reef fishes, using a visual census technique. Estuarine, Coastal and Shelf Science.

[B5239765] Ntiba M. J., Wakwabi E. O., Mwatha G. K., Kimani E., Woitchik A. F. (1993). Species composition and shuttle movement of fish. Dynamics and assessment of Kenyan mangrove ecosystems. Final Report (Project No.TS2-0240-C, GDF) to the European Community.

[B5239839] Pichon M, Stoddart D. R., Younge M. (1971). Comparative studies of the main features of some coral reefs of Madagascar, La Réunion and Mauritius. Regional variation in the Indian Ocean coral reefs.

[B5239876] Pietsch T. W., Smith M. M., Heemstra P. C. (1986). Family No. 102. Antennariidae. Smiths' Sea Fishes.

[B5239920] Ragoonaden S (1997). Impact of sea level rise on Mauritius in Island States at risk: global climate change, development and population. Journal of Coastal Research,.

[B5239948] Rainboth W. J. (1996). Fishes of the Cambodian Mekong. FAO species identification field guide for fishery purposes..

[B5239900] Ramessur R. T. (2013). A Review of Coastal Zone Management Facing Climate Change and Natural Disasters in Mauritius. Journal of Geography & Natural Disasters.

[B5239939] Randall J. E., Allen G. R., Steene R. C. (1990). Fishes of the Great Barrier Reef and Coral Sea.

[B5239930] Randall J. E. (1995). Coastal fishes of Oman.

[B5297394] Roux M. (2013). The diversity and distribution patterns of intertidal fish in the Agulhas bioregion.

[B5239957] Schwarzhans W., Møller P. R. (2007). Review of the Dinematichthyini (Teleostei: Bythitidae) of the Indo-West Pacific. Part III. *Beaglichthys*, *Brosmolus*, *Monothrix* and eight new genera with description of 20 new species.. The Beagle, Records of the Museums and Art Galleries of the Northern Territory.

[B5239779] Sindorf V., Cowburn B., Sluka R. D. (2015). Rocky intertidal fish assemblage of the Watamu Marine National Park, Kenya (Western Indian Ocean). Environmental Biology of Fishes.

[B5239991] Smith M. M., Heemstra P. C., Smith M. M., Heemstra P. C. (1986). Family No. 268. Tetraodontidae. Smiths' Sea Fishes.

[B5239789] Sogard S. M. (1992). Variability in growth rates of juvenile fishes in different estuarine habitats. Marine Ecology Progress Series.

[B5239799] Springer V. G., Spreitzer A. E. (1978). Five new species and a new genus of Indian Ocean blenniid fishes, tribe Salariini, with a key to genera of the tribe. Smithsonian Contributions to Zoology.

[B5239809] Springer V. G., Williams J. T. (1994). The Indo-West Pacific blenniid fish genus *Istiblennius* reappraised : a revision of *Istiblennius*, *Blenniella*, and *Paralticus*, new genus. Smithsonian Contributions to Zoology.

[B5239967] Springer V. G., Fricke R. A. (2000). Description of two new blenniid fish species: *Entomacrodus
lemuria* from the western Indian Ocean and *E.
williamsi* from the western Pacific Ocean. Proceedings of the Biological Society of Washington.

[B5239819] Stepien C. A. (1990). Population structure, diets and biogeographic relationships of a rocky intertidal fish assemblage in central Chile: high levels of herbivory in a temperate system. Bulletin of Marine Science.

[B5240005] Ter Braak C. J.F., Šmilauer P. (2002). CANOCO reference manual and CanoDraw for Windows user’s guide: Software for Canonical Community Ordination (version 4.5).

[B5240035] Thomson Donald A., Lehner Charles E. (1976). Resilience of a rocky intertidal fish community in a physically unstable environment. Journal of Experimental Marine Biology and Ecology.

[B5240045] Thomson J. M., Fischer W., Bianchi G. (1984). Mugilidae. FAO species identification sheets for fishery purposes. Western Indian Ocean (Fishing Area 51).

[B5240014] Tsering L., Pawar H. B., Rayadurga S., Sanaye S. V., Suryavanshi U. (2012). Ichthyofaunal diversity and ecology of intertidal rock pools of Goa, west coast of India. Fishing Chimes.

[B5239610] van der Laan R, Eschmeyer WN, Fricke R (2014). Family-group names of Recent fishes. Zootaxa.

[B5240059] Vasconcelos R. P., Reis-Santos P., Tanner S., Maia A., Latkoczy C., Günther D., Costa M. J., Cabral H. (2008). Evidence of estuarine nursery origin of five coastal fish species along the Portuguese coast through otolith elemental fingerprints. Estuarine, Coastal and Shelf Science.

[B5240087] White G. E., Hose G. C., Brown Culum (2015). Influence of rock-pool characteristics on the distribution and abundance of inter-tidal fishes. Marine Ecology.

[B5240111] Whitehead P. J.P. (1985). FAO Species Catalogue. Vol. 7. Clupeoid fishes of the world (suborder Clupeoidei). An annotated and illustrated catalogue of the herrings, sardines, pilchards, sprats, shads, anchovies and wolf-herrings.

[B5240120] Whitehead P. J.P., Nelson G. J., Wongratana T. (1988). FAO Species Catalogue. Vol. 7. Clupeoid fishes of the world (Suborder Clupeoidei). An annotated and illustrated catalogue of the herrings, sardines, pilchards, sprats, shads, anchovies and wolf-herrings.

[B5240073] Winterbottom R., Smith M. M., Heemstra P. C. (1986). Family No. 227. Congrogadidae. Smiths' sea fishes.

[B5240097] Woodland D. J., Smith M. M., Heemstra P. C. (1986). Family No. 245. Siganidae. Smiths' sea fishes.

